# Choosing the right cell line for renal cell cancer research

**DOI:** 10.1186/s12943-016-0565-8

**Published:** 2016-12-19

**Authors:** Klaudia K. Brodaczewska, Cezary Szczylik, Michal Fiedorowicz, Camillo Porta, Anna M. Czarnecka

**Affiliations:** 1 Department of Oncology with Laboratory of Molecular Oncology, Military Institute of Medicine, Szaserow 128, 04-141 Warsaw, Poland; 2 Department of Experimental Pharmacology, Polish Academy of Science Medical Research Centre, Warsaw, Poland; 3 Department of Medical Oncology, IRCCS San Matteo University Hospital Foundation, Pavia, Italy

**Keywords:** Renal cell cancer, Cell lines, Clear cell RCC, Papillary RCC, In vitro RCC

## Abstract

Cell lines are still a tool of choice for many fields of biomedical research, including oncology. Although cancer is a very complex disease, many discoveries have been made using monocultures of established cell lines. Therefore, the proper use of in vitro models is crucial to enhance our understanding of cancer. Therapeutics against renal cell cancer (RCC) are also screened with the use of cell lines. Multiple RCC in vitro cultures are available, allowing in vivo heterogeneity in the laboratory, but at the same time, these can be a source of errors. In this review, we tried to sum up the data on the RCC cell lines used currently. An increasing amount of data on RCC shed new light on the molecular background of the disease; however, it revealed how much still needs to be done. As new types of RCC are being distinguished, novel cell lines and the re-exploration of old ones seems to be indispensable to create effective in vitro tools for drug screening and more.

**Electronic supplementary material:**

The online version of this article (doi:10.1186/s12943-016-0565-8) contains supplementary material, which is available to authorized users.

## Background

Cell line-based research had a major impact on the development of cancer treatment allowing an innumerable amount of effective drugs to be introduced into practice [1]. Cancer cell lines, especially those in NCI-60 panel, are the first target of preclinical drug screening that enables the quick elimination of ineffective compounds from further preclinical and clinical testing, requiring laboratory animals and patients [2]. Since 1955, the US National Cancer Institute (NCI) has provided screening support to cancer researchers worldwide. Until 1985, the NCI screening program and the selection of compounds for further preclinical and clinical development under NCI auspices had relied predominantly on the in vivo L1210 and P388 murine leukemias and certain other transplantable tumor models. In June of 1984, the author presented to the NCI Division of Cancer Treatment’s Board of Scientific Counselors (BSC) a preliminary concept of a disease-oriented in vitro primary anticancer drug screen as a potential replacement for the P388 in vivo primary screen; after the development of the 60 cell lines panel, it was formally launched in 1990 and is embodied in the present-day screen [3]. The ultimate goal of this disease-oriented screen is to facilitate the discovery of new compounds with potential cell line-specific and/or subpanel-specific antitumor activity [4]. The 60 cell lines of the National Cancer Institute Anticancer Drug Screen (NCI-60) constitute the most extensively characterized in vitro cancer cell model. They have been tested for sensitivity to more than 100,000 potential chemotherapy agents and have been profiled extensively at the DNA, RNA, protein, functional, and pharmacologic levels. Cell lines as a tool in biomedical research have both advantages and disadvantages in comparison with primary cultures and laboratory animals. First, they provide large numbers of cells available for testing, while primary cultures typically have a limited lifespan and require regular access to donors [5]. Importantly, cell lines are a crucial tool in implementing the 3Rs principle of animal research - Replacement, Reduction and Refinement [6] reducing the number of laboratory animals used during primary drug screening. This provides ease and speed of inventions. At the same time established cell lines represent a simplification of natural phenomena, as they are deprived of multilateral relations between different cell populations, microenvironment and responses of the host [7]. This can be partially overcome by culturing cells in complex three-dimensional systems or co-cultures, whaich better mimics natural surroundings in the host, retaining the ease of work and controlled conditions [8, 9]. However, further testing in more relevant biological models is indispensable to proceed to clinical trials but cell line research will still underlaie most of them [10, 11].

Renal cell cancer (RCC) research has greatly benefited from cell line studies. Cell biology studies enable an understanding of RCC biology and translational studies [12–15]. To analyze homogeneous populations of cancer cells and feasibly identify genetic changes (mutations, gene expression) cell lines derived from tumor tissues (nephrectomy and metastasectomy specimens) from patients with renal cell carcinomas are being established [16]. The reported efficacy rate in establishing new cell lines is 75% from fresh and from 35% frozen specimens [16]. In a large study covering 498 successive attempts to establish RCC cell lines, 63 were successful (12%) [17]. The development of new drugs requires an appropriate model for testing; therefore the analysis of the rationale for choosing appropriate cell lines for RCC research is an objective of this review. We focus on popular RCC cell lines, their properties, and usefulness but also note the issues that may be vital for RCC cell line-based research.

### RCC subtypes

Cancer sample (nephrectomy, metastasectomy) derived cell lines are used as in vitro RCC models, and it is important to remember that cell lines are in fact genetic models of their parent tumor histology [18]. Different clinical characteristics and treatment susceptibility are apparent between histotypes of RCC. Cancer cell lines in vitro preserve the unique genetic aberrations of parent tumor from which they were derived, and in long-term culture they acquire additional specific alterations [18]. In culture, cells no longer have easily identifiable morphological characteristics used in the histological classification of tumor specimens. As with primary RCC tumors, the mutation status of cancer including *VLH, cMET* and *TP53* and a general marker immunohistochemistry profile may serve to define the histotype of RCC cell lines (Table 1). Molecular and cell biology researchers using in vitro cell culture as experimental models need to recognize that, like primary cancers, the models used to study diseases genetics, biomarkers, and drug activity/resistance must also be stratified. RCC cell line-based studies are often hampered by a lack of proper annotation of RCC lines. Disease-specific studies need to incorporate cellular and clinical contexts [19, 20]. Surprisingly, many basic pre-clinical RCC studies employing functional research on “renal cancer/renal carcinoma/renal adenocarcinoma/renal cell cancer” cell lines do not analyze the background of the investigated model and analyze different subtypes of RCC together, including wild-type cell lines and those harboring mutations (i.e. VHL) and cell lines of different histotypes [21–23]. The conclusions of such projects may be difficult to interpret, and the value of potential therapeutic targets is rather questionable, as is the true relevance to a particular RCC. Confirming established histotype-specificity markers for RCC cell lines should become the standard in planning and executing experiments on renal carcinoma [23, 24].

**Table 1:** Differentiation of RCC subtypes

Marker	Clear cell RCC	Papillary RCC Type 1	Papillary RCC Type 2	Chromophobe RCC	Oncocytoma	Xp11.2 translocation RCC
VHL mutation	+ (~90%)	-	-	-	-	-
cMET mutation	-	+	-	-	-	-
TP53 mutations	-	-	-	+	-	-
Other mutations	PBRM1 (~50%), BAP1 (~15%), SETD2 (~15%)	NRF2, CUL3	FH	-	Mitochondrial complex I genes	translocations of Xp11.2 (TFE3) or 6p21 (TFEB)
CK8	+/-	+	+	+/-	+	ND
CAIX	+	+/-	+	-	-	+
CAM 5.2	+	+	+	+	+	-
CD10	+	+	+	+/-	+/-	+
CD15	+	+	+	-	+	ND
CK18	+	+	+	+	+	ND
EMA	+	+	+	+	+	-
GST-alpha	+	-	-	-	-	ND
PAX2	+	+	+/-	-	+	+/-
PAX8	+	+	+	+	-	+
RCC Ma	+	+	+/-	-	-	+
VIM	+	+	+	-	-	+
AMACR	-	+	+	-	-	+
CD117	-	-	-	+	+	ND
CK7	-	+/-	-/+	+/-	-	+/-
CK19	-	-	-	-	-	+/-
CK20	-	-	-	-	-	-
c-KIT	-	+/-	+/-	+	+	ND
CLDN7/8	-	-	-	+	+	ND
E-cadherin	-	+	+/-	+	-	+
EpCAM	-	-	-/+	+	+/-	ND
Ksp-cad	-	-	-	-	+	+
PVALB	-	-	-	+	+/-	ND
TFE3	-	-	-	-	-	+
SMA	-	-	-	-	+	ND

Legend: *AMACR* α-methylacyl coenzyme A racemase, *CAIX* carbonic anhydrase IX, *CK7* cytokeratin 7, *CLDN7/8* claudin 7/8, *EMA* epithelial membrane antigen, *GST-alpha* glutathione S-transferase alpha, *EpCAM* epithelial cell adhesion molecule, *Ksp-cad* kidney-specific cadherin, *PVALB* parvalbumin, *RCC Ma* renal cell carcinoma marker, *SMA* smooth muscle action, *TFE3* Transcription factor E3 - transcription factor binding to IGHM enhancer 3, *PAX2/8* paired box gene 2/8, *VIM* vimentin, *ND* no reported data

Major improvements in the pathologic classification of RCC have been reported over last 30 years. The first, known only as renal cell carcinoma, was in the 1960s divided into clear cell and granular histotypes. Currently five traditional and well-defined subtypes of RCC are known: conventional clear cell RCC, papillary (types 1 and 2) RCC, chromophobe RCC, carcinoma of the collecting ducts of Bellini, and unclassified RCC and these subtypes represent the majority of RCC cases diagnosed [25]. Clear cell RCC (ccRCC) is the most common subtype of renal cancer and accounts for approximately 70–75% of cases, so it cannot be assumed that all RCC-derived cell lines represent ccRCC. Papillary RCC (pRCC) is the second most common subtype of RCC and is diagnosed in approximately 10–16% of cases; pRCC is therefore expected among cell lines already in research. In the case of cell lines established in the 1970s or 1980s, histology (based on specific mutations and genetic changes) should be verified before any conclusions of the studies using cell lines are drawn. This particularly applies to new drug developments that are most often histotype specific [26].

In particular, pRCC was characterized in the 1980s as tumors containing more than 75% of papillary structures and not bearing 3p chromosomal loss on the contrary to ccRCC. Later [27], it was found that two different subtypes of papillary tumors may be distinguished (referred as to pRCC Types 1 and 2). Genomic characterization of types 1 and 2 papillary tumors is still incomplete. Inherited forms of types 1 and 2 tumors are referred as to hereditary papillary renal cell and hereditary leiomyomatosis and RCC (HLRCC), respectively. Germline met proto-oncogene (*MET*) and fumarate hydratase (*FH*) alterations are the hallmark of these cancer syndromes, but are infrequent in sporadic cases [28, 29]. It also needs to be underlined that the RCC subtype of clear cell papillary renal cell carcinoma [30] has mixed characteristics of both clear cell and papillary RCC, but possibly some of cell lines may represent this phenotype.

Chromophobe RCC (chRCC) is the third most common RCC subtype, and it was described for the first time in the mid-1980s. Additionally, rare histologic RCC subtypes were discovered in the 1990s and 2000s and include collecting duct carcinoma, medullary RCC, translocation RCC, and mucinous tubular and spindle-cell RCC. Recently, even more new subtypes have been described- hybrid oncocytic chromophobe tumor, mucinous tubular and spindle cell carcinoma, multilocular cystic clear cell RCC of low malignant potential, carcinoma associated with neuroblastoma, and renal medullary carcinoma [25, 31, 32]. Nevertheless, one should also not forget RCC types that were approved in 2013 by the International Society of Urological Pathology (ISUP) Vancouver Consensus Statement including five more epithelial tumor subtypes: the micropthalmia (MiT) family translocation RCCs (Xp11 translocation RCC), tubulocystic RCC, acquired cystic disease-associated, RCC clear cell tubulopapillary RCC, and hereditary leiomyomatosis–RCC syndrome-associated tumors. Next, three RCC subtypes were given provisional status- thyroid-like follicular carcinoma of kidney, succinate dehydrogenase B deficiency-associated RCC, and anaplastic lymphoma kinase translocation RCC [31, 32].

To distinguish RCC subtypes, genetic analysis may be employed. The von Hippel-Lindau (VHL) gene is known to be most often mutated in renal cell carcinoma of clear cell type (ccRCC) in up to 90% of sporadic ccRCC cases [33] and multiple surprising and contradictory reports on the VHL gene status in common RCC cell lines have been published (Additional file 1: Table S1). Moreover recently three other tumor suppressor genes PBRM1 (mutated in ~50%), BAP1 (~15%), and SETD2 (~15%) were defined as specific for the ccRCC subtype. PBRM1, also known as BAF180 or Polybromo, is a member of the PBAF SWI/SNF chromatin remodeling complex. *VHL, PBRM1, BAP1* and *SETD2* are allocated on chromosome 3p. BAP 1 is a BRCA1- associated protein-1 (ubiquitin carboxy-terminal hydrolase). Mutations in *BAP1* and *PBRM1* in ccRCC tend to be mutually exclusive [34].

This in vivo heterogeneity of RCC should be mimicked in vitro; a wide panel of cell lines with different characteristics is needed to provide us with a tool for both basic and applied research.

### RCC cell lines used in research

**Fig. 1:**
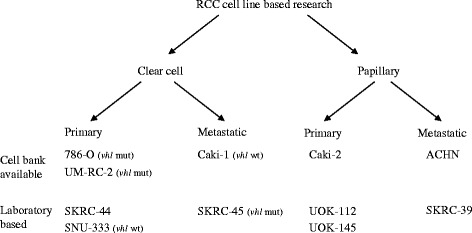
Classical RCC cell lines as models of different RCC subtypes and disease stage

The number of available RCC cell lines is impressive: more than 20 cell lines are widely used- deposited in cell banks- and dozens of others were established and used for research in selected laboratories (Fig. 1) [16]. The most popular RCC cell lines are delivered by ATCC and other certified cell banks (Additional file 1: Table S1). Most cell lines were established between the mid-1970s till the late 1980s, when subtypes of RCC including clear cell, papillary, or chromophobe RCC were not yet distinguished; therefore, all the subtypes may be represented among RCC cell lines. Many researchers currently refer generally to RCC or renal carcinoma when describing their laboratory model. Although this is true, it regrettably narrows conclusions that can be drawn from their research, thus limiting the translational potential of in vitro studies. However, a growing amount of data can help to categorize cell lines established before 1995 into correct RCC subtypes as the cell lines genetic profile is analyzed and cells lines are characterized for markers of particular RCC subtypes with multiple methods including immunohistochemistry (IHC), gene sequencing, and xenografted tumors histology analysis [35, 36]. The proper initial molecular characterization of cell lines is indispensable to provide later in vitro tools to study genetic and cellular events underlying carcinogenesis, disease progression, and/or drug activity [23, 37–39]. Often, information on original patients- cell line donors- is fragmentary and more precise characteristics of established cell lines come with time, thanks to cell biology and genetic studies [28, 33]. Most recently, interesting cell lines representing novel RCC subtypes have been established, including NCCFH1 for hereditary papillary renal cell carcinoma type 2 [40] or the S-TFE cell line for Xp11 translocation renal cell carcinoma [41]. In Additional file 1: Table S1, we collected various information available for over 60 cell lines- including the source of starting material, basic genomic data, and database tools that might be useful in designing RCC cell line-based experiments.

### ACHN cell line

The ACHN cell line represents an uncertain RCC histotype (Fig. 1). It was established from pleural effusion and models metastatic disease. In early xenograft studies, tumors were described as poorly differentiated clear cell [42] however, a more recent genomic comparison suggests papillary characteristics of ACHN [43]. Moreover, this cell line harbors a *c-met* polymorphism that is specific for papillary RCC [44]. Chromosome aberrations in ACHN cells also resemble those of papillary tumors [45]. Yet, gene expression analysis revealed similarities to clear cell tumors, especially when concerning the *MYC* pathway [29]. No mutations in VHL and HIF-1α mRNA in ACHN cell line, could confirm non-clear cell histology [46].

### A-498 cell line

A-498 is a “classical” RCC cell line belonging to the NCI-60 panel and is therefore widely used in cancer research. Mutated *vhl* [46, 47] would suggest a clear cell subtype; however, a study by [48] detected no mutation. In addition, [49] claimed that A-498 is of papillary origin, as xenografts exhibited such histology. To some extent this observation was confirmed by chromosome 8 and *MYC* analysis [29]. Nonetheless, this issue was not significantly recognized by scientific community and most researchers use it as a model of ccRCC [47, 50, 51] with rare exceptions [52].

### 786-O cell line

786-O, established as one of the first RCC cell lines, has many characteristics of ccRCC and is used most commonly in RCC-focused research (Fig. 1). The 786-O RCC line is defective in *VHL* expression, as it harbors mutated *VHL* [53, 54] with altered HIF and VEGF (Vascular endothelial growth factor) pathways [46] and gives rise to clear cell tumors in nude mice [55, 56]. In this RCC model the vast majority (122/160) of genes induced by hypoxia in wt-VHL transfected 786-O (*VHL* ^+^) cells are not significantly up-regulated in *VHL* mutated 786-O cells, confirming that the loss of *VHL* is not equivalent to hypoxic exposure and that in RCC, the *VHL* tumor suppressor has a distinct role from its activity in the hypoxia-inducible pathway [57]. Interestingly, side populations (SPs) of higher tumorigenicity were observed in this cell line, proving its usefulness in cancer stem cell studies [58, 59]. Surface receptors also confirm the ccRCC phenotype of 786-O cells, as these cells are positive for CD10 [60] and vimentin [61]. These cells produce high levels of VEGF [46] as well, which is characteristic of ccRCC. This cell line can be used to model bone metastasis in RCC- 786-O cells injected into nude mice, both directly to the tibia or to the cardiac ventricle, cause bone destruction and vascularization [62, 63]. A subline derived from such metastatic tumors can be cultured in vitro in 3D systems that retain bone metastasis characteristics [64].

### Caki-1 cell line

Caki-1 is a widespread model line of metastatic ccRCC (Fig. 1). While harboring wild-type *vhl*, it was shown to produce tumors of clear cell histology in nude mice, both by the total population and SP cells [65]. High VEGF production [66] (especially in hypoxic conditions [56]) in those cells is also a hallmark of ccRCC. Interestingly, this cell line was also proposed as a model system of proximal tubule epithelium, as in culture, cells can form a polarized layer with morphological, physiological and biochemical characteristics of functional, well-differentiated kidney tissue [67].

### Caki-2 cell line

Caki-2 was established from a primary tumor of the kidney. This cell line was primarily defined as the ccRCC cell line (Fig. 1) that expresses wild-type pVHL but does not express HIF-2α. However, a low expression of HIF-1α is detected in this cell line for unknown reasons [46]. The recent evaluation of tumors formed by Caki-2 in nude mouse in orthotopic and sub-cutaneous implantations revealed cystic papillary tumors with microvilli and microfilaments, few mitochondria, lysosomes or lipid droplets, and multilamellar bodies [68–70]. Although the Caki-2 cell line has been treated as a model for primary ccRCC, a growing amount of data suggests that it is a cell line of papillary RCC. These cells express high levels of MET and LRRK2 [71] and harbor chromosome 8 aberrations [29] characteristic ofr papillary RCC. Interestingly, reports on *VHL* gene status of this cell line are inconsistent– some researchers detected no mutations [46], while others [47, 48] reported mutations in α-domain of VHL, which may imply the clear cell histology of Caki-2. Such misleading observations have led to non-uniform use of this cell line–it is now widely used both in clear cell RCC [66, 72, 73] and papillary RCC [69–71, 74] research. Finally, it is generally described as clear cell RCC by most cell banks, which may lead to misinterpretation.

### 769-P cell line

The 769-P cell line, established along with 786-O by [75], harbors mutated *vhl* and secretes high levels of VEGF, suggesting a ccRCC phenotype (Additional file 1: Table S1 for ref). These cells contain a SP of higher tumorigenicity that can create tumors even after serial passage in nude mice retaining their original histological characteristics [76]. However, the validity of this cell line in xenograft studies is limited; injected subcutaneously [56] or *i.v.* [55], they failed to form tumors in some models.

### RCC4 cell line

Another interesting cell line is RCC4, a *vhl* mutant [77, 78] derived from a primary tumor widely used as a model for VHL-dependant mechanisms, witha commercially available counterpart cell line with restored wild-type gene [79]. It is used [80–83] for both in vitro and in vivo experiments, as RCC4 cells are tumorigenic in nude mice. Unfortunately, no data on the original patient can be found; supposedly the cells were obtained by Prof. C.H.C.M. Buys, Department of Medical Genetics, University of Groningen, Groningen, the Netherlands.

### SMKT-R cell lines

SMKT-R1, SMKT-R2, SMKT-R3 and SMKT-R4 are cell lines established in Sapporo Medical College from primary lesions of RCC from xenotransplantable tumors at passage 2 or 1 in nude mice [84]. *Vhl* is mutated in cell lines SMKT-R2 and SMKT-R3 [48], and in SMKT-R2 and SMKT-R3 HIF-α proteins are expressed [85]. SMKT-R3 original tumors are characterized as papillary type and granular cell subtype, and the level of secreted VEGF is lower than in SMKT-R2 line [86] confirming the non-ccRCC histology of this cell line. Research on the SMKT-R cell lines confirms that RCC cell lines can retain the histology of the original tumor after in vitro culture and passage in nude mice.

### Memorial Sloan Kettering Cancer Centre cell line collection

The metastatic dissemination of cancer may be partially recapitulated in vitro with analysis of multiple metastatic loci and metastasis-derived cell lines. This in vivo heterogeneity of tumors can be mimicked in vitro with use of a panel of cell lines in the place of multiple biopsy derived samples [87]. The Memorial Sloan Kettering Cancer Centre provides an RCC repository with over 30 primary tumor-derived and 15 metastatic tumor-derived cell lines (SK-RC panel) collected between 1972 and 1987 [17]. The MSKCC panel covers cell lines obtained from tumors that developed in the most common RCC metastases loci, including the adrenal glands (SK-RC-45), lymph nodes (SK-RC-18, SK-RC-26b), lungs (SK-RC-26a SK-RC-31, SK-RC-38 SK-RC-54), bones (SK-RC-42, SK-RC-46), soft tissue (SK-RC-17, SK-RC-39), and the brain (SK-RC-9, SK-RC-13). This collection consists of samples with different features; however, the molecular characterization of particular cell lines is still incomplete (Additional file 1: Table S1). The SK-RC-45 line was used to study immune responses in RCC and the induction of T cell apoptosis [88, 89], while SK-RC-42 was shown to contain cancer stem cells (“CSCs”) [90]. These cell lines express either HIF-2α only (SKRC-21, SKRC-17) or both HIF-1α and HIF-2α (SKRC-7, SKRC-10, SKRC-52) [91].

### Laboratory specific RCC cell line collections

3Thirty cell lines from primary tumors as well as four lines from metastatic tissues taken from 31 patients were established in the National Cancer Institute in Bethesda (UOK 101–131 cell lines). Sixteen of the cell lines were derived from tumors composed predominantly of clear cell RCC, three were granular cell RCCs, and one papillary type (UOK112). The remaining tumors were of mixed types including clear and granular cells, clear cell + granular and sarcomatoid cells (UOK 105, 117, 119, 123 and 127), or clear cell and papillary (OUK 120). Cell line phenotypes, including morphology, in vitro growth characterization, and tumorigenicity in nude mice were determined for these cell lines [16]. Another seven RCC cell lines were established in the Korean Cell Line Bank of Cancer Research Center and Cancer Research Institute. In particular, five cell lines were derived from clear cell RCC (SNU-228, -267, -328, -349, and -1272), one from granular RCC (SNU-482), and one from mixed clear and granular RCC (SNU-333). The mutational status of cell lines was confirmed for von Hippel-Lindau (VHL), p53, TGF-beta type II receptor (TGF-betaRII), hMSH2, and hMLH1 genes [92]. More recently, Chinese-origin cell lines named NRCC from the primary ccRCC and MRCC from the metastatic ccRCC were established from the primary tumor of a 49-year-old male ccRCC patient and the metastatic tumor of a 62-year-old male with ccRCC. The morphology of cell lines along with the doubling times, colony formation rates, invasion assay, anchorage-independent growth, cytogenetic characteristics, and expression of CD105, CD133, CD44, CD24, CD56, CD99, and CD74 markers as well as N-cadherin, E-cadherin, and vimentin were described and have shown that NRCC cells displayed more epithelial characteristics, while MRCC cells are mesenchymal-like [93].

### RCC cancer stem cell cultures

Specific subpopulations of cancer cells are available for culture. In particular cancer stem cells referred to as tumor-initiating cells are currently becoming available. In particular, donor specific kidney cancer cells derived from primary tumors cultured in stem cell-promoting media are enriched in CSCs (Promab cat. No. CF100107, Celprogen cat. No. 36117-44). The role of CSCs in RCC has been reviewed elsewhere [94], as they are potential treatment targets [95]. They are putatively tumor-initiating cells that promote disease development and progression and may be distinguished using different approaches. CSCs can be discriminated based on their unique features; in different cancers, in comparison to other tumor cells, they have elevated aldehyde dehydrogenase (ALDH) activity, increased glycolysis and glycine/serine metabolism or low concentrations of reactive oxygen species and ATP, the ability to form spheres, and a reduced level of proliferation. Such functional characteristics can be used to selectively isolate subpopulations of cancer cells [96–99]. Moreover, due to the increased action of ABC transporters, CSCs are more resistant to drug treatment, which enables the separation of CSCs from other tumor cells based on the increased efflux of ABC-dependent dyes (Rhodamine123 or Hoechst33342) as a dye-negative side population (SP) [98, 100]. In RCC, CD105 and CD133 surface markers are also suspected to identify cells with stem properties [101, 102]. CSCs express genes typical for multi/pluri-potent cells; Oct4, Sox-2, Nanog, and Bmi-1 [98, 103] and are shown to have increased tumorigenicity and clonogenicity. In stable cell cultures of RCC cells, cancer stem cells have been identified, which primarily reflects the unexpected heterogeneity of cell culture in vitro. In 769-P, ACHN, Caki-1, SMKT-R2, and SMKT-R3 cell lines, the SP of Hoechst33342 negative cells was shown to express CSC properties [76, 104]. The ACHN line CSC subpopulation is Hoechst33342-negative and ALDH-positive [98], while Caki-2 CSCs are also ALDH-positive and form spheres [99]. The 786-O cell line is also shown to bear the CSC subpopulation, as confirmed by Rh123 fluorescent intensity based isolation [105]. At the same time, SK-RC-42 cells cultured in spheres have features of CSCs [90]. However, methods in CSCs research are not yet uniform, and some contradictory data complicate the explanation of their role in RCC. In particular, [105] showed that Rh123low cells show stronger CSC properties than the Rh123high population that could be suspected to group stem cells.

### Normal renal proximal tubule cells

Primary cultures of normal human epithelial cells of proximal origin derived from the renal cortex have been shown to present very homogeneous morphology in vitro [106]. If normal tissue originating from the same donor is not available, RPTEC cells are used as the control cell line for the comparative analysis of cancer and normal renal cells [57]. RPTEC cells are human renal proximal tubule epithelial cells that are derived from normal or diseased (e. g. diabetic) donors [107]. As primary cells, they have a limited lifespan, but usually can reach at least eight passages maintaining typical epithelial, cobblestone morphology and the expression of epithelial and renal markers like E-Cadherin, CK, or ZO-1 [108, 109]. These cells are also available as immortalized lines: HK-2 and RPTEC/hTERT. The former was transduced with human papilloma virus (HPV 16) E6/E7 genes [110] while the latter ectopically expresses the catalytic subunit of telomerase (TERT) [111]. These modifications enable the cells to be cultured continuously constituting a convenient control for RCC cell lines. Many studies show that the cells are genomically stable and most functional characteristics remain typical for RPTEC [111]. RPTECs are used to model basic kidney functions and renal diseases. Nephotoxicity [112], the efflux of drugs [113], responses to environmental toxicants [114] or renin-angiotensin system (RAS) signaling [115] can be studied with the use of these cells. In RPTECs, the steady-state amount of VHL protein is strictly regulated by the cell density, and the cellular VHL content is more than 100-fold higher in dense cultures than in sparse cultures [116].

Total kidney isolates are also available (e. g. Applied StemCell, cat. no. ASE-5186); after the dissociation of a healthy renal sample, all cells are frozen which allows various cell types to be cultured without direct access to donors.

Another widely used non-cancer renal cell line is the HEK293 cell line. Generated in 1977 by the viral transformation of human embryonal kidney cells, [117] HEK293 cell line is a widely used expression tool [118]. Although studies show that these cells have many features of neuronal cells [119, 120], they are still used as a model for kidney physiology [121, 122]. Therefore, they may be used as convenient controls in RCC in vitro research as well, but caution is needed for interpretation [73, 123].

### RCC cell lines specific for bone metastasis-oriented research

RCC cell lines have also been developed as tools to study specific phenomena in RCC. In particular, a model to study the biology of the bone metastasis of renal cell carcinoma has been established. This cell line induced osteolytic lesions in nude mice after injecting into the tibia. RBM1 cell line cells expressed high levels of cytokines involved in osteoclast activation and bone resorption- parathyroid hormone-related peptide, interleukin-6, and macrophage colony-stimulating factor. Moreover, cells were confirmed to express EGFR and c-MET [124]. SK-RC-42 and SK-RC-46 also represent bone metastasis-derived cell lines [17], as well as the CRBM-1990 cell line [125], while ACHN and 786-O cells transplanted into the left ventricle establish bone metastases [126, 127]. If studied after injection, bone metastasis-derived 786-O cells (Bo-786-O) compared to parental 786-O cells or cells that localized in the liver or lymph nodes had significantly overexpressed cadherin-11, but not CXCR4, HIF-1α, VEGF, angiopoeitin-1, Tie2, c-MET, PTHrP, IL-6 or RANKL [128]. Sunitinib prevents the growth of ACHN cells in a bone metastatic model. The number of osteoclasts in sunitinib-treated ACHN-bearing mice is significantly lower than that in non-treated mice [126].

### Other RCC cell lines

RCC subtypes less frequent than clear cell and papillary are even more difficult to study in vitro as specimens are obtained rarely. Recently, several cell lines were developed to model HLRCC; a rare genetic disorder that manifests by cutaneous and uterine leiomyomas and kidney tumors. Metastatic NCCFH1, UOK262, and primary UOK268 cell lines are FH deficient and can serve as models for hereditary papillary type 2 RCC [40, 129, 130]. An in vitro model for Xp11.2 translocation carcinoma has recently been established as well. S-TFE cell line is tumorigenic in nude mice and has fused TFE3 and ASPL genes [41].

### Large scale molecular data and RCC cell lines

Some RCC cell lines, mostly present in NCI-60, were used in whole genome analyses. Mutational, copy variant, and expression analyses available for cells lines are provided as links to databases in Additional file 1: Table S1. The Cancer Genome Atlas (“TGCA”) provided much valuable information on RCC characteristics that can now be used in cell line-based research. TGCA analyses identified *vhl*, *pbrm1*, *bap1*, *setd2, kdm5c, pten* and *mtor* as the most frequently mutated genes in ccRCC [131]. *vhl* mutant cell lines are easily accessible; 786-O, 769-P, RCC4 (with reintroduced *vhl* control cell line available), are the most widely studied models for VHL role in RCC. Apart from gene mutation, *vhl* promoter region methylation often occurs in ccRCC samples which effects in no protein expression. Such a phenomenon was reported for SK-RC-54, 769-P, and A-704 [132].

Classical cell lines were recently verified for PBRM1 expression status and confirmed ACHN- positive, 786-O– positive, and A-704– negative. The ACHN cell line expresses the protein, but harbors a heterozygous nonsense mutation, while 786-O, 769-P, Caki-1, and A-498 express wild-type PBRM1 [133, 134]. The PBRM1 mutation is also reported in Caki-2 and A-704 lines; loss-of-function gene mutations in A-704 and the deletion in exon 17 of the PBRM1 gene in Caki-2 [135] results in no protein expression [133, 134, 136]. Mutations have also been detected in OS-RC-2 and RCC-ER (see Additional file 1: Table S1 for ref) cell lines; however, the former was reported to express PBRM1 protein [133]. The strong and moderate expression of PBRM1 was also confirmed in the next 16 cell lines, including normal human embryonic renal cell lines (HEK 293 and 293T) and human renal proximal tubular epithelial cells (HK2) as well as cancer cell lines- 769-P, A-498, KC12, Caki-1, SW156, and SLR21-26 [133]. The knockdown of PBRM1 in cells with wild-type gene increased the proliferation, migration and colony formation abilities [137], supporting this gene’s important role in RCC progression, as the loss of PBRM1 was correlated with a worse disease outcome in patients [133].

BAP1 mutants are available as UM-RC-6, 769-P, and SN12C cell lines [138]; however, 769-P cells still produce the protein [139]. The reintroduction of the wild-type gene reduced cell proliferation and sensitized cells to treatment, and it was proposed that BAP1 is a tumor suppressor, as gene loss is associated with patients with higher-grade RCC [139]. SETD2 mutations have also been detected in A-498, A-704, Caki-1, and RCC-ER [140–142], PTEN mutations in 786-O, and OS-RC-2, while no mutated RCC cell lines could be found for KDM5C. An mTOR mutation was found for SNU349 and RCC-ER cell lines only (see Additional file 1: Table S1 for ref). Studies on SETD2-defective RCC cells proved that the mutation of this gene affects DNA repair and may correlate with in vivo disease progression [143].

Chromosome alterations are also common in ccRCC with the loss of the 3p chromosome (containing *vhl*, *pbrm1*, *bap1,* and *setd2*), 14q loss (*hif1a*), or 5q gain being the most frequent [131]. However, the chromosome analysis of RCC cell lines is not readily available; a 5q gain was observed in most RCC cell lines tested by [144] apart from A-498. Chromosome 3p loss was reported in several UOK RCC cell lines: UOK108, 121, 125, and 127 [145]. Simultaneous losses in 3p and 14q were observed in 769-P, 786-O, A-704, and Caki-1 [146, 147].

In the case of pRCC, TCGA indicated *met*, *setd2*, *nf2*, *kdm6a*, *smarcb1* and *fat1*, *bap1*, *pbrm1*, *stag2*, *nfe2l2*, *tp53* genes being the most frequently mutated among patient samples [148]. The UOK112 cell line derived from pRCC patient [16] was studied in the context of HGF/MET signaling; however, no data on *met* status could be found [149]. Interestingly, Caki-1 harbors a mutation in *met* (COSMIC database). As mentioned above, ACHN cells have a MET polymorphism (by some also referred to as a mutation [150]), but protein was detected and can be phosphorylated [151]. This cell line was also shown to contain an NF2 mutation, together with SN12C, while Caki-1, A-704, 769-P, TK10, 786-O, A-498, and OS-RC-2 were confirmed to be wild-type for NF2 [142]. A KDM6A mutation was reported for the SN12C cell line only (COSMIC database), but no alterations in the methylation of gene promoter was observed in 5 other cell lines (786-O, 769-P, A-498, ACHN, and Caki-1) by [152]. A FAT1 mutation was found in Caki-1, OS-RC-2, SN12C, RCC-FG2, and TK10 (COSMIC database) but no functional analysis of this gene in in vitro RCC was reported.

Chromosome aberrations present in pRCC include chromosomes 7 and 17 gains and 9p loss [148]. In the case of cell lines, Caki-2 and ACHN cells show a gain in genes located on chromosomes 7 and 17 and 9p loss, which may be an additional clue confirming the papillary origin of these cells [146, 153].

TP53 tumor-suppressor mutations, present in 50% of tumor cases in general, are less frequent in RCC (around 20% cases [154]), but confirmed in 786-O, A-498 (COSMIC and CCLE databases),,SN12C, TK10 [155] and reported as wild-type in ACHN, Caki-1, and Caki-2 [156]. Varied expression of the protein is visible in cell lines, which corresponds with in vivo data, as p53 over-expression occurs in later stages of the disease [157] and to some extent correlates with poor prognosis [158]. A relatively high expression of p53 was observed in ACHN, Caki-2, UOK121, and UM-RC-6 and low in A-498 (although mutated) [159, 160]. Other genes, established by TCGA studies to be often mutated in RCC, have been less frequently studied with the use of RCC cell lines, and no detailed information on gene status could be found.

### RCC cell lines in xenograft studies

The clinical translability of cell line experiments relies on valid animal models. They provide complex platforms for studying oncogenesis and the effectiveness of therapeutic approaches and, enable the verification of cell lines’ tumorigenicity. While tumorigenic cells can be implanted into the same species (allografts) or another species (xenografts), we will focus on immunocompromised experimental animals that are injected with human cell lines. The wide application of this approach in oncology studies followed the discovery of two groups of immunodeficient animals: “nude” mice [161] and later severe combined immunodeficient (severe combined immunodeficiency; SCID) mice [162].

Athymic nude mice are hairless, an effect of the Foxn1^nu^ (Forkhead box protein N1) mutation, but more importantly they lack a thymus and are T-cell deficient but produce functional B-cells [163]. This is not the case with SCID mice that have a single nucleotide polymorphism (Prkdc^scid^) within the DNA-dependent protein kinase of the catalytic polypeptide Prkdc gene. This mutation affects both T and B lymphocytes [162]. SCID rodents display less pronounced immunoreactivity than athymic nude mice to implanted cancer cells that results in greater receptivity to tumor xenotransplantation [164]. Recently, several promising transgenic models have become available, including humanized NSG mice [165]. Still, most of the available data on xenografted RCC cell lines comes from the athymic nude mice model.

The nature of the outcome of animal experiments strictly relies on the place and route of cell inoculation. Ectopic tumor xenograft models employ subcutaneous (s.c), intraperitenoeal (i.p.), intravenous (i.v.) or intramuscular (i.m.) implantation of tumor cells. While advantages are the approach’s simplicity and reproducibility, an obvious disadvantage of this approach is the non-physiologic growth location. An alternative approach, the orthotopic xenograft model, involves the implantation of a tumor into the originating tissue site of the cancer in rodents. In the case of RCC, the widely accepted implantation site is the renal subcapsule [166, 167]. It is believed that orthotopic tumor implantation more closely simulates the microenvironment of the original tumor [168]. However, orthotopic xenograft models have some additional disadvantages that could be crucial in RCC research: they are more technically challenging and may have highly variable tumor take rates and growth rates; they could also result in significant animal morbidity due to the surgical implantation of tumor cells [168]. Importantly, the monitoring of tumor growth is also more challenging in orthotopic models. All these difficulties have led to subcutaneous ectopic implantation being the most widely used approach in RCC cell lines animal research.

Ectopic xenograft models of athymic nude mice with various genetic backgrounds have been extensively used for studying established RCC lines, including 769-P, 786-O, Caki-1, SK-RC-38, SK-RC-42, SK-RC-44, SK-RC-45, SK-RC-46, and others [17, 55, 169–173]. As summarized in Additional file 1: Table S1, most of the cell lines described in this article were proven to be tumorigenic in nude mice. The implanted tumors normally become palpable within 5 days and reach a volume of 100 mm^3^ in 2 weeks [169–171]. Implanted established cell lines xenografted into nude mice preserve essentially the same histology as the primary tumors [174]. In addition, orthotopic xenotransplantation of Caki-2 into nude mouse produces tumors that closely resemble histology of human RCC.

Generally, xenografted tumors are considered as not producing metastasis in most cases. Sharkey and Fogh [175] studied 106 malignant human tumor lines and observed metastasis in only approximately 1%. Such factors as tumor size and growth rate and age and sex of the host mouse appear unrelated to metastasis [167]. However, the incidence of metastasis is increased in SCID rodents [176]. Moreover, the route of injection into nude mice affects the metastasis incidence. Naito et al. [167] indicated that even in highly metastatic cells, i.v. injection did not yield significant metastasis, but the injection of cells orthotopically into the renal subcapsule resulted in extensive metastasis to the lungs and in all peritoneal organs. However, Strube et al. [127] were successful in generating remarkable metastasis to the bone by inoculating human 786-O cells into the left cardiac ventricle of athymic nude mice. Caki-1, A-498, and 786-O injected intravenously produced metastasis very rarely in nude mice as well as in NOD SCID [177].

Finally, some cell lines fail to be tumorigenic in nude mouse; 769-P, SK-RC-7, TK 10, TK 164 UM-RC-6, or UOK108 [17, 145, 172, 178, 179]. Tumorigenicity in animal models depends on the intrinsic capability of the tumor line employed. However, these results should be interpreted with some caution, as potential failure could also rely on the specific strain or age of animals [180]. Still, precisely designed and controlled xenograft experiments remain a powerful and useful tool in RCC research.

### Future of RCC cell line based research

Despite the undisputed profits that translational medicine has gained from research conducted on cell lines, several concerns have arisen regarding the extent to which cell line results can be trusted [181–183]. Despite its limitations, as a cell line does not strictly resemble an in vivo tumor [184], the proper description of widely used cell lines is indispensable. Currently, new RCC cell lines are still being established. The isolation of cells from normal and tumor samples can be performed with various protocols [107, 185]. These can be used either for primary cultures [186] or to establish new cell lines [125]. Unfortunately, newly established cell lines rarely receive recognition in the RCC community. Most stick to widely known cell lines, such as: 786-O, 769-P, Caki-1, or ACHN. At the same time, the interpretation of research on some of these cell lines seems to be challenging and may require re-interpretation. As described above, data on particular cell lines, like most scientific information [187], grows cumulatively, and at times we need to redefine primary assumptions (e. g., cancer type) and consequently interpreted phenomena. For instance, if the Caki-2 cell line is indeed a model of papillary RCC, reviewing past studies may shed new light on the molecular background of this particular cancer subtype. As papillary RCC research is rather underrepresented [188], this would be of value for scientists and patients.

Cancer is a complex disease shaped by changes in cell functions, but also intrinsic signaling inside the tumor and extrinsic interactions with other cells of the host as well as various components of the local microenvironment. Such a multiplex disorder is very difficult to interpret, and cell line research does not always reflect cancer diversity. The limitations of the validity of cell line cultures apply also to RCC. To increase the usefulness of cell lines for RCC discovery, complex in vitro models have been designed. It was shown that samples from RCC patients cultured as 3D organoids create structures that closely mimic in vivo tumor, serving as a useful model for personalized drug screening [186]. 3D cultures for cancer studies is a hot topic that has been reviewed recently in different aspects [189, 190]. Such structures can also be created by cell lines; 3D cultures have been shown to be a better model of in vivo mechanisms in cancer than standard 2D techniques [191]. The use of established cell lines would allow high throughput platforms to be created that are useful for effective drug screening [190, 192]. Such methods are of need in RCC research; however, to create a valid in vitro screening tool properly characterized cell lines are indispensable.

## Conclusions

When conducting research using established cell lines, one should carefully study the data on their establishment and subsequently available characteristics. Thorough data on original patients has not been was provided consistently in prior research; however, modern molecular analysis helps to characterize cell line features. Certain RCC specimens are underrepresented, such as: papillary and chromophobe RCC, metastatic ccRCC (especially tissues that are most often affected in vivo; lungs, bones), and early stage tumors. When establishing new cell lines, it is essential to follow the best available guidelines, e. g. reviewed elsewhere [193]. Thorough data on original patients’ tissue in terms of histological and molecular characteristics should be collected, and subsequent cell line stability should be monitored. A panel of well-characterized RCC cell lines that reflect in vivo heterogeneity in terms of different subtypes, grades, and drug resistance would arm us with a screening tool to test new therapy strategies and understand the molecular background of RCC subtypes.

### Take-home message

- Molecular profiling of RCC cell lines is not always available, which may limit the clinical translation of in vitro research; back checking of model cell lines for typical RCC features is needed
- research on numerous cell lines with relation to healthy tissues can increase the clinical value of RCC cell line research; familiarity with their features is indispensable to draw accurate conclusions
- Additional RCC cell lines and culture models are needed to mirror in vivo heterogeneity
- When establishing new RCC cell lines thorough characterization of OP data and subsequent culture are indispensable to create a useful in vitro tool

## Additional file

Additional file 1: Characteristics of renal cell cancer cell lines including their origin, histology, culture conditions, and molecular characteristics. (DOCX 355 kb)

## Abbreviations

ALDH: Aldehyde dehydrogenase
ccRCC: Clear cell renal cell carcinoma
FH: Fumarate hydratase
Foxn1: Forkhead box protein N1
MET: Met proto-oncogene
PDGFR: Platelet-derived growth factor receptors
pRCC: Papillary renal cell carcinoma
RAS: Renin-angiotensin system
RCC: Renal cell carcinoma
RPTEC: Renal proximal tubule cells
SCID: Severe combined immunodeficiency
TERT: Telomerase
VEGF: Vascular endothelial growth factor
VHL: Von Hippel-Lindau

## References

[1] Sharma SV, Haber DA, Settleman J (2010). Cell line-based platforms to evaluate the therapeutic efficacy of candidate anticancer agents. Nat Rev Cancer.

[2] Shoemaker RH (2006). The NCI60 human tumour cell line anticancer drug screen. Nat Rev Cancer.

[3] Stinson SF, Alley MC, Kopp WC, Fiebig HH, Mullendore LA, Pittman AF, Kenney S, Keller J, Boyd MR (1992). Morphological and immunocytochemical characteristics of human tumor cell lines for use in a disease-oriented anticancer drug screen. Anticancer Res.

[4] Monks A, Scudiero D, Skehan P, Shoemaker R, Paull K, Vistica D, Hose C, Langley J, Cronise P, Vaigro-Wolff A (1991). Feasibility of a high-flux anticancer drug screen using a diverse panel of cultured human tumor cell lines. J Natl Cancer Inst.

[5] Zieba J, Ksiazkiewcz M, Janik K, Banaszczyk M, Peciak J, Piaskowski S, Lipinski M, Olczak M, Stoczynska-Fidelus E, Rieske P (2015). Sensitivity of neoplastic cells to senescence unveiled under standard cell culture conditions. Anticancer Res.

[6] Russell Wms BK (1992). The principles of humane experimental technique.

[7] Arul M, Roslani AC, Ng CL, Cheah SH (2014). Culture of low passage colorectal cancer cells and demonstration of variation in selected tumour marker expression. Cytotechnology.

[8] Benien P, Swami A (2014). 3D tumor models: history, advances and future perspectives. Future Oncol.

[9] Berg EL, Hsu YC, Lee JA (2014). Consideration of the cellular microenvironment: physiologically relevant co-culture systems in drug discovery. Adv Drug Deliv Rev.

[10] Hutchinson L, Kirk R (2011). High drug attrition rates--where are we going wrong?. Nat Rev Clin Oncol.

[11] Tuveson D, Hanahan D (2011). Translational medicine: Cancer lessons from mice to humans. Nature.

[12] Siemeister G, Weindel K, Mohrs K, Barleon B, Martiny-Baron G, Marmé D (1996). Reversion of deregulated expression of vascular endothelial growth factor in human renal carcinoma cells by von Hippel-Lindau tumor suppressor protein. Cancer Res.

[13] Wilhelm S, Chien D-S (2002). BAY 43-9006: preclinical data. Curr Pharm Des.

[14] Kane RC, Farrell AT, Saber H, Tang S, Williams G, Jee JM, Liang C, Booth B, Chidambaram N, Morse D (2006). Sorafenib for the treatment of advanced renal cell carcinoma. Clin Cancer Res.

[15] Amundson SA, Do KT, Vinikoor LC, Lee RA, Koch-Paiz CA, Ahn J, Reimers M, Chen Y, Scudiero DA, Weinstein JN (2008). Integrating global gene expression and radiation survival parameters across the 60 cell lines of the National Cancer Institute Anticancer Drug Screen. Cancer Res.

[16] Anglard P, Trahan E, Liu S, Latif F, Merino MJ, Lerman MI, Zbar B, Linehan WM (1992). Molecular and cellular characterization of human renal cell carcinoma cell lines. Cancer Res.

[17] Ebert T, Bander NH, Finstad CL, Ramsawak RD, Old LJ (1990). Establishment and characterization of human renal cancer and normal kidney cell lines. Cancer Res.

[18] Greshock J, Nathanson K, Martin AM, Zhang L, Coukos G, Weber BL, Zaks TZ (2007). Cancer cell lines as genetic models of their parent histology: analyses based on array comparative genomic hybridization. Cancer Res.

[19] Czarnecka AM, Kornakiewicz A, Kukwa W, Szczylik C (2014). Frontiers in clinical and molecular diagnostics and staging of metastatic clear cell renal cell carcinoma. Future Oncol.

[20] Czarnecka AM, Kukwa W, Kornakiewicz A, Lian F, Szczylik C (2014). Clinical and molecular prognostic and predictive biomarkers in clear cell renal cell cancer. Future Oncol.

[21] Mizumoto A, Yamamoto K, Nakayama Y, Takara K, Nakagawa T, Hirano T, Hirai M (2015). Induction of epithelial-mesenchymal transition via activation of epidermal growth factor receptor contributes to sunitinib resistance in human renal cell carcinoma cell lines. J Pharmacol Exp Ther.

[22] Hutson TE, Al-Shukri S, Stus VP, Lipatov ON, Shparyk Y, Bair AH, Rosbrook B, Andrews GI, Vogelzang NJ. Axitinib Versus Sorafenib in First-Line Metastatic Renal Cell Carcinoma: Overall Survival From a Randomized Phase III Trial. Clin Genitourin Cancer 2016. doi:10.1016/j.clgc.2016.05.008. [Epub ahead of print] 10.1016/j.clgc.2016.05.008 27498023

[23] Anglesio MS, Wiegand KC, Melnyk N, Chow C, Salamanca C, Prentice LM, Senz J, Yang W, Spillman MA, Cochrane DR (2013). Type-specific cell line models for type-specific ovarian cancer research. PLoS One.

[24] Tani T, Laitinen L, Kangas L, Lehto VP, Virtanen I (1995). Expression of E- and N-cadherin in renal cell carcinomas, in renal cell carcinoma cell lines in vitro and in their xenografts. Int J Cancer.

[25] Crumley SM, Divatia M, Truong L, Shen S, Ayala AG, Ro JY (2013). Renal cell carcinoma: Evolving and emerging subtypes. World J Clin Cases.

[26] Escudier B, Porta C, Schmidinger M, Algaba F, Patard JJ, Khoo V, Eisen T, Horwich A (2014). Renal cell carcinoma: ESMO Clinical Practice Guidelines for diagnosis, treatment and follow-up. Ann Oncol.

[27] Delahunt B, Eble JN (1997). Papillary renal cell carcinoma: a clinicopathologic and immunohistochemical study of 105 tumors. Mod Pathol.

[28] Schmidt L, Duh FM, Chen F, Kishida T, Glenn G, Choyke P, Scherer SW, Zhuang Z, Lubensky I, Dean M (1997). Germline and somatic mutations in the tyrosine kinase domain of the MET proto-oncogene in papillary renal carcinomas. Nat Genet.

[29] Furge KA, Dykema K, Petillo D, Westphal M, Zhang Z, Kort EJ, Teh BT (2007). Combining differential expression, chromosomal and pathway analyses for the molecular characterization of renal cell carcinoma. Can Urol Assoc J.

[30] Liddell H, Mare A, Heywood S, Bennett G, Chan HF (2015). Clear cell papillary renal cell carcinoma: a potential mimic of conventional clear cell renal carcinoma on core biopsy. Case Rep Urol.

[31] Srigley JR, Delahunt B, Eble JN, Egevad L, Epstein JI, Grignon D, Hes O, Moch H, Montironi R, Tickoo SK (2013). The International Society of Urological Pathology (ISUP) Vancouver Classification of Renal Neoplasia. Am J Surg Pathol.

[32] Shuch B, Amin A, Armstrong AJ, Eble JN, Ficarra V, Lopez-Beltran A, Martignoni G, Rini BI, Kutikov A (2015). Understanding pathologic variants of renal cell carcinoma: distilling therapeutic opportunities from biologic complexity. Eur Urol.

[33] Nickerson ML, Jaeger E, Shi Y, Durocher JA, Mahurkar S, Zaridze D, Matveev V, Janout V, Kollarova H, Bencko V (2008). Improved identification of von Hippel-Lindau gene alterations in clear cell renal tumors. Clin Cancer Res.

[34] Brugarolas J (2013). PBRM1 and BAP1 as novel targets for renal cell carcinoma. Cancer J.

[35] Van Bergen NJ, Wood JP, Chidlow G, Trounce IA, Casson RJ, Ju WK, Weinreb RN, Crowston JG (2009). Recharacterization of the RGC-5 retinal ganglion cell line. Invest Ophthalmol Vis Sci.

[36] Boonstra JJ, van der Velden AW, Beerens EC, van Marion R, Morita-Fujimura Y, Matsui Y, Nishihira T, Tselepis C, Hainaut P, Lowe AW (2007). Mistaken identity of widely used esophageal adenocarcinoma cell line TE-7. Cancer Res.

[37] Domcke S, Sinha R, Levine DA, Sander C, Schultz N (2013). Evaluating cell lines as tumour models by comparison of genomic profiles. Nat Commun.

[38] van Staveren WC, Solis DY, Hebrant A, Detours V, Dumont JE, Maenhaut C (2009). Human cancer cell lines: Experimental models for cancer cells in situ? For cancer stem cells?. Biochim Biophys Acta.

[39] Lauvrak SU, Munthe E, Kresse SH, Stratford EW, Namlos HM, Meza-Zepeda LA, Myklebost O (2013). Functional characterisation of osteosarcoma cell lines and identification of mRNAs and miRNAs associated with aggressive cancer phenotypes. Br J Cancer.

[40] Perrier-Trudova V, Huimin BW, Kongpetch S, Huang D, Ong P, Le Formal A, Poon SL, Siew EY, Myint SS, Gad S (2015). Fumarate Hydratase-deficient Cell Line NCCFH1 as a New In Vitro Model of Hereditary Papillary Renal Cell Carcinoma Type 2. Anticancer Res.

[41] Hirobe M, Masumori N, Tanaka T, Kitamura H, Tsukamoto T (2013). Establishment of an ASPL-TFE3 renal cell carcinoma cell line (S-TFE). Cancer Biol Ther.

[42] Korhonen M, Sariola H, Gould VE, Kangas L, Virtanen I (1994). Integrins and laminins in human renal carcinoma cells and tumors grown in nude mice. Cancer Res.

[43] Hakimi AA, Chevinsky M, Hsieh JJ, Sander C, Sinha R (2014). Mp23-11 Genomic Comparison of Renal Cell Carcinoma Cell Lines to Human Tumors. J Urol.

[44] Schmidt L, Junker K, Nakaigawa N, Kinjerski T, Weirich G, Miller M, Lubensky I, Neumann HP, Brauch H, Decker J (1999). Novel mutations of the MET proto-oncogene in papillary renal carcinomas. Oncogene.

[45] Kovacs G, Fuzesi L, Emanual A, Kung HF (1991). Cytogenetics of papillary renal cell tumors. Genes Chromosomes Cancer.

[46] Shinojima T, Oya M, Takayanagi A, Mizuno R, Shimizu N, Murai M (2007). Renal cancer cells lacking hypoxia inducible factor (HIF)-1alpha expression maintain vascular endothelial growth factor expression through HIF-2alpha. Carcinogenesis.

[47] Kucejova B, Pena-Llopis S, Yamasaki T, Sivanand S, Tran TA, Alexander S, Wolff NC, Lotan Y, Xie XJ, Kabbani W (2011). Interplay between pVHL and mTORC1 pathways in clear-cell renal cell carcinoma. Mol Cancer Res.

[48] Ashida S, Nishimori I, Tanimura M, Onishi S, Shuin T (2002). Effects of von Hippel-Lindau gene mutation and methylation status on expression of transmembrane carbonic anhydrases in renal cell carcinoma. J Cancer Res Clin Oncol.

[49] Lovell M, Lott ST, Wong P, El-Naggar A, Tucker S, Killary AM (1999). The genetic locus NRC-1 within chromosome 3p12 mediates tumor suppression in renal cell carcinoma independently of histological type, tumor microenvironment, and VHL mutation. Cancer Res.

[50] Robb VA, Karbowniczek M, Klein-Szanto AJ, Henske EP (2007). Activation of the mTOR signaling pathway in renal clear cell carcinoma. J Urol.

[51] Campbell L, Al-Jayyoussi G, Gutteridge R, Gumbleton N, Griffiths R, Gumbleton S, Smith MW, Griffiths DF, Gumbleton M (2013). Caveolin-1 in renal cell carcinoma promotes tumour cell invasion, and in co-operation with pERK predicts metastases in patients with clinically confined disease. J Transl Med.

[52] Hsu RJ, Ho JY, Cha TL, Yu DS, Wu CL, Huang WP, Chu P, Chen YH, Chen JT, Yu CP (2012). WNT10A plays an oncogenic role in renal cell carcinoma by activating WNT/beta-catenin pathway. PLoS One.

[53] Ding XF, Zhou J, Hu QY, Liu SC, Chen G (2015). The tumor suppressor pVHL down-regulates never-in-mitosis A-related kinase 8 via hypoxia-inducible factors to maintain cilia in human renal cancer cells. J Biol Chem.

[54] Iliopoulos O, Kibel A, Gray S, Kaelin WG (1995). Tumour suppression by the human von Hippel-Lindau gene product. Nat Med.

[55] Kozlowski JM, Fidler IJ, Campbell D, Xu ZL, Kaighn ME, Hart IR (1984). Metastatic behavior of human tumor cell lines grown in the nude mouse. Cancer Res.

[56] Miyake M, Goodison S, Lawton A, Zhang G, Gomes-Giacoia E, Rosser CJ (2013). Erythropoietin is a JAK2 and ERK1/2 effector that can promote renal tumor cell proliferation under hypoxic conditions. J Hematol Oncol.

[57] Jiang Y, Zhang W, Kondo K, Klco JM, St Martin TB, Dufault MR, Madden SL, Kaelin WG, Nacht M (2003). Gene expression profiling in a renal cell carcinoma cell line: dissecting VHL and hypoxia-dependent pathways. Mol Cancer Res.

[58] Lin Y, Yang Z, Xu A, Dong P, Huang Y, Liu H, Li F, Wang H, Xu Q, Wang Y (2015). PIK3R1 negatively regulates the epithelial-mesenchymal transition and stem-like phenotype of renal cancer cells through the AKT/GSK3beta/CTNNB1 signaling pathway. Sci Rep.

[59] Zhang L, Jiao M, Wu K, Li L, Zhu G, Wang X, He D, Wu D (2014). TNF-alpha induced epithelial mesenchymal transition increases stemness properties in renal cell carcinoma cells. Int J Clin Exp Med.

[60] Boysen G, Bausch-Fluck D, Thoma CR, Nowicka AM, Stiehl DP, Cima I, Luu VD, von Teichman A, Hermanns T, Sulser T (2012). Identification and functional characterization of pVHL-dependent cell surface proteins in renal cell carcinoma. Neoplasia.

[61] Ho MY, Tang SJ, Chuang MJ, Cha TL, Li JY, Sun GH, Sun KH (2012). TNF-alpha induces epithelial-mesenchymal transition of renal cell carcinoma cells via a GSK3beta-dependent mechanism. Mol Cancer Res.

[62] Strube A, Stepina E, Mumberg D, Scholz A, Hauff P, Käkönen S-M (2010). Characterization of a new renal cell carcinoma bone metastasis mouse model. Clin Exp Metastasis.

[63] Xie C, Schwarz EM, Sampson ER, Dhillon RS, Li D, O’Keefe RJ, Tyler W (2012). Unique angiogenic and vasculogenic properties of renal cell carcinoma in a xenograft model of bone metastasis are associated with high levels of vegf-a and decreased ang-1 expression. J Orthop Res.

[64] Pan T, Fong ELS, Martinez M, Harrington DA, Lin S-H, Farach-Carson MC, Satcher RL (2015). Three-dimensional (3D) culture of bone-derived human 786-O renal cell carcinoma retains relevant clinical characteristics of bone metastases. Cancer Lett.

[65] Lichner Z, Saleh C, Subramaniam V, Seivwright A, Prud'homme GJ, Yousef GM (2015). miR-17 inhibition enhances the formation of kidney cancer spheres with stem cell/tumor initiating cell properties. Oncotarget.

[66] Liu YH, Lin CY, Lin WC, Tang SW, Lai MK, Lin JY (2008). Up-Regulation of Vascular Endothelial Growth Factor-D Expression in Clear Cell Renal Cell Carcinoma by CD74: A Critical Role in Cancer Cell Tumorigenesis. J Immunol.

[67] Glube N, Giessl A, Wolfrum U, Langguth P (2007). Caki-1 cells represent an in vitro model system for studying the human proximal tubule epithelium. Nephron Exp Nephrol.

[68] Pulkkanen KJ, Parkkinen JJ, Kettunen MI, Kauppinen RA, Lappalainen M, Ala-Opas MY, Yla-Herttuala S (2000). Characterization of a new animal model for human renal cell carcinoma. In Vivo.

[69] Pulkkanen KJ, Parkkinen JJ, Laukkanen JM, Kettunen MI, Tyynela K, Kauppinen RA, Ala-Opas MY, Yla-Herttuala S (2001). HSV-tk gene therapy for human renal cell carcinoma in nude mice. Cancer Gene Ther.

[70] Furge KA, Chen J, Koeman J, Swiatek P, Dykema K, Lucin K, Kahnoski R, Yang XJ, Teh BT (2007). Detection of DNA copy number changes and oncogenic signaling abnormalities from gene expression data reveals MYC activation in high-grade papillary renal cell carcinoma. Cancer Res.

[71] Looyenga BD, Furge KA, Dykema KJ, Koeman J, Swiatek PJ, Giordano TJ, West AB, Resau JH, Teh BT, MacKeigan JP (2011). Chromosomal amplification of leucine-rich repeat kinase-2 (LRRK2) is required for oncogenic MET signaling in papillary renal and thyroid carcinomas. Proc Natl Acad Sci U S A.

[72] Blondeau JJ, Deng M, Syring I, Schrodter S, Schmidt D, Perner S, Muller SC, Ellinger J (2015). Identification of novel long non-coding RNAs in clear cell renal cell carcinoma. Clin Epigenetics.

[73] Zaravinos A, Pieri M, Mourmouras N, Anastasiadou N, Zouvani I, Delakas D, Deltas C (2014). Altered metabolic pathways in clear cell renal cell carcinoma: A meta-analysis and validation study focused on the deregulated genes and their associated networks. Oncoscience.

[74] Roos FC, Evans AJ, Brenner W, Wondergem B, Klomp J, Heir P, Roche O, Thomas C, Schimmel H, Furge KA (2011). Deregulation of E2-EPF ubiquitin carrier protein in papillary renal cell carcinoma. Am J Pathol.

[75] Williams RD, Elliott AY, Stein N, Fraley EE (1976). In vitro cultivation of human renal cell cancer. I. Establishment of cells in culture. In Vitro.

[76] Huang B, Huang YJ, Yao ZJ, Chen X, Guo SJ, Mao XP, Wang DH, Chen JX, Qiu SP (2013). Cancer stem cell-like side population cells in clear cell renal cell carcinoma cell line 769P. PLoS One.

[77] Harten SK, Esteban MA, Shukla D, Ashcroft M, Maxwell PH (2011). Inactivation of the von Hippel-Lindau tumour suppressor gene induces Neuromedin U expression in renal cancer cells. Mol Cancer.

[78] Razorenova OV, Finger EC, Colavitti R, Chernikova SB, Boiko AD, Chan CK, Krieg A, Bedogni B, LaGory E, Weissman IL (2011). VHL loss in renal cell carcinoma leads to up-regulation of CUB domain-containing protein 1 to stimulate PKC{delta}-driven migration. Proc Natl Acad Sci U S A.

[79] Maxwell PH, Wiesener MS, Chang GW, Clifford SC, Vaux EC, Cockman ME, Wykoff CC, Pugh CW, Maher ER, Ratcliffe PJ (1999). The tumour suppressor protein VHL targets hypoxia-inducible factors for oxygen-dependent proteolysis. Nature.

[80] Harada H, Itasaka S, Zhu Y, Zeng L, Xie X, Morinibu A, Shinomiya K, Hiraoka M (2009). Treatment regimen determines whether an HIF-1 inhibitor enhances or inhibits the effect of radiation therapy. Br J Cancer.

[81] Esteban MA, Tran MG, Harten SK, Hill P, Castellanos MC, Chandra A, Raval R, O’Brien TS, Maxwell PH (2006). Regulation of E-cadherin expression by VHL and hypoxia-inducible factor. Cancer Res.

[82] Raval RR, Lau KW, Tran MG, Sowter HM, Mandriota SJ, Li JL, Pugh CW, Maxwell PH, Harris AL, Ratcliffe PJ (2005). Contrasting properties of hypoxia-inducible factor 1 (HIF-1) and HIF-2 in von Hippel-Lindau-associated renal cell carcinoma. Mol Cell Biol.

[83] Zhang H, Gao P, Fukuda R, Kumar G, Krishnamachary B, Zeller KI, Dang CV, Semenza GL (2007). HIF-1 inhibits mitochondrial biogenesis and cellular respiration in VHL-deficient renal cell carcinoma by repression of C-MYC activity. Cancer Cell.

[84] Miyao N, Tsukamoto T, Kumamoto Y (1989). Establishment of three human renal cell carcinoma cell lines (SMKT-R- SMKT-R-2, and SMKT-R-3) and their characters. Urol Res.

[85] Tanaka T, Torigoe T, Hirohashi Y, Sato E, Honma I, Kitamura H, Masumori N, Tsukamoto T, Sato N (2014). Hypoxia-inducible factor (HIF)-independent expression mechanism and novel function of HIF prolyl hydroxylase-3 in renal cell carcinoma. J Cancer Res Clin Oncol.

[86] Tochizawa S, Masumori N, Yanai Y, Ohmoto Y, Yabuuchi Y, Tsukamoto T (2008). Antitumor effects of a combination of interferon-alpha and sorafenib on human renal carcinoma cell lines. Biomed Res.

[87] Gerlinger M, Rowan AJ, Horswell S, Larkin J, Endesfelder D, Gronroos E, Martinez P, Matthews N, Stewart A, Tarpey P (2012). Intratumor heterogeneity and branched evolution revealed by multiregion sequencing. N Engl J Med.

[88] Kudo D, Rayman P, Horton C, Cathcart MK, Bukowski RM, Thornton M, Tannenbaum C, Finke JH (2003). Gangliosides expressed by the renal cell carcinoma cell line SK-RC-45 are involved in tumor-induced apoptosis of T cells. Cancer Res.

[89] Das T, Sa G, Paszkiewicz-Kozik E, Hilston C, Molto L, Rayman P, Kudo D, Biswas K, Bukowski RM, Finke JH (2008). Renal cell carcinoma tumors induce T cell apoptosis through receptor-dependent and receptor-independent pathways. J Immunol.

[90] Zhong Y, Guan K, Guo S, Zhou C, Wang D, Ma W, Zhang Y, Li C, Zhang S (2010). Spheres derived from the human SK-RC-42 renal cell carcinoma cell line are enriched in cancer stem cells. Cancer Lett.

[91] Sjolund J, Johansson M, Manna S, Norin C, Pietras A, Beckman S, Nilsson E, Ljungberg B, Axelson H (2008). Suppression of renal cell carcinoma growth by inhibition of Notch signaling in vitro and in vivo. J Clin Invest.

[92] Shin KH, Ku JL, Kim WH, Lee SE, Lee C, Kim SW, Park JG (2000). Establishment and characterization of seven human renal cell carcinoma cell lines. BJU Int.

[93] Tan X, He S, Han Y, Yu Y, Xiao J, Xu D, Wang G, Du Y, Chang W, Yin J (2013). Establishment and characterization of clear cell renal cell carcinoma cell lines with different metastatic potential from Chinese patients. Cancer Cell Int.

[94] Myszczyszyn A, Czarnecka AM, Matak D, Szymanski L, Lian F, Kornakiewicz A, Bartnik E, Kukwa W, Kieda C, Szczylik C (2015). The Role of Hypoxia and Cancer Stem Cells in Renal Cell Carcinoma Pathogenesis. Stem Cell Rev.

[95] Czarnecka M, Cezary Szczylik A (2013). Renal Cell Carcinoma Cancer Stem Cells as Therapeutic Targets. Curr Signal Transduction Ther.

[96] Hasmim M, Bruno S, Azzi S, Gallerne C, Michel JG, Chiabotto G, Lecoz V, Romei C, Spaggiari GM, Pezzolo A (2016). Isolation and characterization of renal cancer stem cells from patient-derived xenografts. Oncotarget.

[97] Lucarelli G, Galleggiante V, Rutigliano M, Vavallo A, Ditonno P, Battaglia M (2015). Isolation and characterization of cancer stem cells in renal cell carcinoma. Urologia.

[98] Ueda K, Ogasawara S, Akiba J, Nakayama M, Todoroki K, Sanada S, Suekane S, Noguchi M, Matsuoka K, Yano H (2013). Aldehyde dehydrogenase 1 identifies cells with cancer stem cell-like properties in a human renal cell carcinoma cell line. PLoS One.

[99] Wang L, Park P, La Marca F, Than KD, Lin CY (2015). BMP-2 inhibits tumor-initiating ability in human renal cancer stem cells and induces bone formation. J Cancer Res Clin Oncol.

[100] Khan MI, Czarnecka AM, Helbrecht I, Bartnik E, Lian F, Szczylik C (2015). Current approaches in identification and isolation of human renal cell carcinoma cancer stem cells. Stem Cell Res Ther.

[101] Bussolati B, Bruno S, Grange C, Ferrando U, Camussi G (2008). Identification of a tumor-initiating stem cell population in human renal carcinomas. FASEB J.

[102] Kim K, Park BH, Ihm H, Kim KM, Jeong J, Chang JW, Cho YM (2011). Expression of stem cell marker CD133 in fetal and adult human kidneys and pauci-immune crescentic glomerulonephritis. Histol Histopathol.

[103] Wang D, Lu P, Zhang H, Luo M, Zhang X, Wei X, Gao J, Zhao Z, Liu C (2014). Oct-4 and Nanog promote the epithelial-mesenchymal transition of breast cancer stem cells and are associated with poor prognosis in breast cancer patients. Oncotarget.

[104] Nishizawa S, Hirohashi Y, Torigoe T, Takahashi A, Tamura Y, Mori T, Kanaseki T, Kamiguchi K, Asanuma H, Morita R (2012). HSP DNAJB8 controls tumor-initiating ability in renal cancer stem-like cells. Cancer Res.

[105] Lu J, Cui Y, Zhu J, He J, Zhou G, Yue Z (2013). Biological characteristics of Rh123 stem-like cells in a side population of 786-O renal carcinoma cells. Oncol Lett.

[106] Detrisac CJ, Sens MA, Garvin AJ, Spicer SS, Sens DA (1984). Tissue culture of human kidney epithelial cells of proximal tubule origin. Kidney Int.

[107] Valente MJ, Henrique R, Costa VL, Jeronimo C, Carvalho F, Bastos ML, de Pinho PG, Carvalho M (2011). A rapid and simple procedure for the establishment of human normal and cancer renal primary cell cultures from surgical specimens. PLoS One.

[108] Giron-Michel J, Azzi S, Khawam K, Mortier E, Caignard A, Devocelle A, Ferrini S, Croce M, Francois H, Lecru L (2012). Interleukin-15 plays a central role in human kidney physiology and cancer through the gammac signaling pathway. PLoS One.

[109] Baer PC, Bereiter-Hahn J, Schubert R, Geiger H (2006). Differentiation status of human renal proximal and distal tubular epithelial cells in vitro: Differential expression of characteristic markers. Cells Tissues Organs.

[110] Ryan MJ, Johnson G, Kirk J, Fuerstenberg SM, Zager RA, Torok-Storb B (1994). HK- an immortalized proximal tubule epithelial cell line from normal adult human kidney. Kidney Int.

[111] Wieser M, Stadler G, Jennings P, Streubel B, Pfaller W, Ambros P, Riedl C, Katinger H, Grillari J, Grillari-Voglauer R (2008). hTERT alone immortalizes epithelial cells of renal proximal tubules without changing their functional characteristics. Am J Physiol Renal Physiol.

[112] Jenkinson SE, Chung GW, van Loon E, Bakar NS, Dalzell AM, Brown CD (2012). The limitations of renal epithelial cell line HK-2 as a model of drug transporter expression and function in the proximal tubule. Pflugers Arch - Eur J Physiol.

[113] Tramonti G, Romiti N, Norpoth M, Chieli E (2001). P-glycoprotein in HK-2 proximal tubule cell line. Ren Fail.

[114] Simon BR, Wilson MJ, Wickliffe JK (2014). The RPTEC/TERT1 cell line models key renal cell responses to the environmental toxicants, benzo[a]pyrene and cadmium. Toxicol Rep.

[115] Handa RK (2001). Characterization and Signaling of the AT4 Receptor in Human Proximal Tubule Epithelial (HK-2) Cells. J Am Soc Nephrol.

[116] Baba M, Hirai S, Kawakami S, Kishida T, Sakai N, Kaneko S, Yao M, Shuin T, Kubota Y, Hosaka M (2001). Tumor suppressor protein VHL is induced at high cell density and mediates contact inhibition of cell growth. Oncogene.

[117] Graham FL, Smiley J, Russell WC, Nairn R (1977). Characteristics of a human cell line transformed by DNA from human adenovirus type 5. J Gen Virol.

[118] Thomas P, Smart TG (2005). HEK293 cell line: a vehicle for the expression of recombinant proteins. J Pharmacol Toxicol Methods.

[119] Madhusudana SN, Sundaramoorthy S, Ullas PT (2010). Utility of human embryonic kidney cell line HEK-293 for rapid isolation of fixed and street rabies viruses: comparison with Neuro-2a and BHK-21 cell lines. Int J Infect Dis.

[120] Shaw G, Morse S, Ararat M, Graham FL (2002). Preferential transformation of human neuronal cells by human adenoviruses and the origin of HEK 293 cells. FASEB J.

[121] Ashokkumar B, Vaziri ND, Said HM (2006). Thiamin uptake by the human-derived renal epithelial (HEK-293) cells: cellular and molecular mechanisms. Am J Physiol Renal Physiol.

[122] Waly MI, Al Moundhri MS, Ali BH (2011). Effect of Curcumin on Cisplatin-and Oxaliplatin-Induced Oxidative Stress in Human Embryonic Kidney (HEK) 293 Cells. Ren Fail.

[123] De Araujo Junior RF, Leitao Oliveira AL, de Melo Silveira RF, de Oliveira Rocha HA, de Franca Cavalcanti P, de Araujo AA (2015). Telmisartan induces apoptosis and regulates Bcl-2 in human renal cancer cells. Exp Biol Med (Maywood).

[124] Weber KL, Pathak S, Multani AS, Price JE (2002). Characterization of a renal cell carcinoma cell line derived from a human bone metastasis and establishment of an experimental nude mouse model. J Urol.

[125] Avnet S, Cenni E, Granchi D, Perut F, Amato I, Battistelli L, Brandi ML, Giunti A, Baldini N (2004). Isolation and characterization of a new cell line from a renal carcinoma bone metastasis. Anticancer Res.

[126] Maita S, Yuasa T, Tsuchiya N, Mitobe Y, Narita S, Horikawa Y, Hatake K, Fukui I, Kimura S, Maekawa T (2012). Antitumor effect of sunitinib against skeletal metastatic renal cell carcinoma through inhibition of osteoclast function. Int J Cancer.

[127] Weber KL1, Pathak S, Multani AS, Price JE. Characterization of a renal cell carcinoma cell line derived from a human bone metastasis and establishment of an experimental nude mouse model. J Urol 2002;168(2):774-9. 12131367

[128] Satcher RL, Pan T, Cheng CJ, Lee YC, Lin SC, Yu G, Li X, Hoang AG, Tamboli P, Jonasch E (2014). Cadherin-11 in renal cell carcinoma bone metastasis. PLoS One.

[129] Yang Y, Valera V, Sourbier C, Vocke CD, Wei M, Pike L, Huang Y, Merino MA, Bratslavsky G, Wu M (2012). A novel fumarate hydratase-deficient HLRCC kidney cancer cell line, UOK268: a model of the Warburg effect in cancer. Cancer Genet.

[130] Yang Y, Valera VA, Padilla-Nash HM, Sourbier C, Vocke CD, Vira MA, Abu-Asab MS, Bratslavsky G, Tsokos M, Merino MJ (2010). UOK 262: Fumarate Hydratase (-/-) Hereditary Leiomyomatosis Renal Cell Carcinoma: In Vitro and In Vivo Model of an Aberrant Energy Metabolic Pathway in Human Cancer. Cancer Genet Cytogenet.

[131] TCGAR Network (2013). Comprehensive molecular characterization of clear cell renal cell carcinoma. Nature.

[132] Ricketts CJ, Morris MR, Gentle D, Shuib S, Brown M, Clarke N, Wei W, Nathan P, Latif F, Maher ER (2013). Methylation profiling and evaluation of demethylating therapy in renal cell carcinoma. Clin Epigenetics.

[133] Pawlowski R, Muhl SM, Sulser T, Krek W, Moch H, Schraml P (2013). Loss of PBRM1 expression is associated with renal cell carcinoma progression. Int J Cancer.

[134] Brugarolas J (2013). PBRM1 and BAP1 as novel targets for renal cell carcinoma. Cancer J.

[135] Chowdhury B, Porter EG, Stewart JC, Ferreira CR, Schipma MJ, Dykhuizen EC (2016). PBRM1 Regulates the Expression of Genes Involved in Metabolism and Cell Adhesion in Renal Clear Cell Carcinoma. PLoS One.

[136] Forbes SA, Bindal N, Bamford S, Cole C, Kok CY, Beare D, Jia M, Shepherd R, Leung K, Menzies A (2011). COSMIC: mining complete cancer genomes in the Catalogue of Somatic Mutations in Cancer. Nucleic Acids Res.

[137] Varela I, Tarpey P, Raine K, Huang D, Ong CK, Stephens P, Davies H, Jones D, Lin ML, Teague J (2011). Exome sequencing identifies frequent mutation of the SWI/SNF complex gene PBRM1 in renal carcinoma. Nature.

[138] Peña-Llopis S, Vega-Rubín-de-Celis S, Liao A, Leng N, Pavía-Jiménez A, Wang S, Yamasaki T, Zhrebker L, Sivanand S, Spence P (2012). BAP1 loss defines a new class of renal cell carcinoma. Nat Genet.

[139] Piva F, Santoni M, Matrana MR, Satti S, Giulietti M, Occhipinti G, Massari F, Cheng L, Lopez-Beltran A, Scarpelli M, Principato G, Cascinu S, Montironi R (2015). BAP1, PBRM1 and SETD2 in clear-cell renal cell carcinoma: molecular diagnostics and possible targets for personalized therapies. Expert Rev Mol Diagn.

[140] Feng C, Sun Y, Ding G, Wu Z, Jiang H, Wang L, Ding Q, Wen H. PI3Kβ Inhibitor TGX221 Selectively Inhibits Renal Cell Carcinoma Cells with Both VHL and SETD2 mutations and Links Multiple Pathways. Scientific Reports, Published online: 8 April 2015; | doi: 10.1038/srep09465 2015. 10.1038/srep09465 PMC5396071 25853938

[141] Duns G, van den Berg E, van Duivenbode I, Osinga J, Hollema H, Hofstra RMW, Kok K (2010). Histone Methyltransferase Gene SETD2 Is a Novel Tumor Suppressor Gene in Clear Cell Renal Cell Carcinoma. Cancer Res.

[142] Dalgliesh GL, Furge K, Greenman C, Chen L, Bignell G, Butler A, Davies H, Edkins S, Hardy C, Latimer C (2010). Systematic sequencing of renal carcinoma reveals inactivation of histone modifying genes. Nature.

[143] Kanu N, Grönroos E, Martinez P, Burrell RA, Goh XY, Bartkova J, Maya-Mendoza A, Mistrík M, Rowan AJ, Patel H (2015). SETD2 loss-of-function promotes renal cancer branched evolution through replication stress and impaired DNA repair. Oncogene.

[144] Li L, Shen C, Nakamura E, Ando K, Signoretti S, Beroukhim R, Cowley GS, Lizotte P, Liberzon E, Bair S (2013). SQSTM1 is a Pathogenic Target of 5q Copy Number Gains in Kidney Cancer. Cancer Cell.

[145] Reiter RE, Anglard P, Liu S, Gnarra JR, Linehan WM (1993). Chromosome 17p deletions and p53 mutations in renal cell carcinoma. Cancer Res.

[146] Strefford JC, Stasevich I, Lane TM, Lu YJ, Oliver T, Young BD (2005). A combination of molecular cytogenetic analyses reveals complex genetic alterations in conventional renal cell carcinoma. Cancer Genet Cytogenet.

[147] Alimov A, Kost-Alimova M, Liu J, Li C, Bergerheim U, Imreh S, Klein G, Zabarovsky ER (2000). Combined LOH/CGH analysis proves the existence of interstitial 3p deletions in renal cell carcinoma. Oncogene.

[148] Network TCGAR: Comprehensive Molecular Characterization of Papillary Renal-Cell Carcinoma. http://dxdoiorg/101056/NEJMoa1505917 2016. 10.1056/NEJMoa1505917 PMC4775252 26536169

[149] Lee YH, Morrison BL, Bottaro DP (2014). Synergistic Signaling of Tumor Cell Invasiveness by Hepatocyte Growth Factor and Hypoxia*. J Biol Chem.

[150] Lee YH, Apolo AB, Agarwal PK, Bottaro DP (2014). Characterization of HGF/Met Signaling in Cell Lines Derived From Urothelial Carcinoma of the Bladder. Cancers.

[151] Gibney GT, Aziz SA, Camp RL, Conrad P, Schwartz BE, Chen CR, Kelly WK, Kluger HM (2013). c-Met is a prognostic marker and potential therapeutic target in clear cell renal cell carcinoma. Ann Oncol.

[152] Ibragimova I, Maradeo ME, Dulaimi E, Cairns P (2013). Aberrant promoter hypermethylation of PBRM1, BAP1, SETD2, KDM6A and other chromatin-modifying genes is absent or rare in clear cell RCC. Epigenetics.

[153] Shen C, Beroukhim R, Schumacher SE, Zhou J, Chang M, Signoretti S, Kaelin WG (2011). Genetic and Functional Studies Implicate HIF1α as a 14q Kidney Cancer Suppressor Gene. Cancer Discov.

[154] Girgin C, Tarhan H, Hekimgil M, Sezer A, Gurel G (2001). P53 mutations and other prognostic factors of renal cell carcinoma. Urol Int.

[155] Abaan OD, Polley EC, Davis SR, Zhu YJ, Bilke S, Walker RL, Pineda M, Gindin Y, Jiang Y, Reinhold WC (2013). The Exomes of the NCI-60 Panel: a Genomic Resource for Cancer Biology and Systems Pharmacology. Cancer Res.

[156] Tomita Y, Bilim V, Kawasaki T, Takahashi K, Okan I, Magnusson KP, Wiman KG (1996). Frequent expression of Bcl-2 in renal-cell carcinomas carrying wild-type p53. Int J Cancer.

[157] Noon AP, Vlatković N, Polański R, Maguire M, Shawki H, Parsons K, Boyd MT (2010). p53 and MDM2 in Renal Cell Carcinoma: Biomarkers for Disease Progression and Future Therapeutic Targets?. Cancer.

[158] Zigeuner R, Ratschek M, Rehak P, Schips L, Langner C (2004). Value of p53 as a prognostic marker in histologic subtypes of renal cell carcinoma: a systematic analysis of primary and metastatic tumor tissue. Urology.

[159] Warburton HE, Brady M, Vlatković N, Linehan WM, Parsons K, Boyd MT (2005). p53 Regulation and Function in Renal Cell Carcinoma. Cancer Res.

[160] Galbán S, Martindale JL, Mazan-Mamczarz K, de Silanes López I, Fan J, Wang W, Decker J, Gorospe M (2003). Influence of the RNA-Binding Protein HuR in pVHL-Regulated p53 Expression in Renal Carcinoma Cells. Mol Cell Biol.

[161] Flanagan SP (1966). ‘Nude’, a new hairless gene with pleiotropic effects in the mouse. Genet Res.

[162] Bosma MJ, Carroll AM (1991). The SCID mouse mutant: definition, characterization, and potential uses. Annu Rev Immunol.

[163] Morton CL, Houghton PJ (2007). Establishment of human tumor xenografts in immunodeficient mice. Nat Protoc.

[164] Taghian A, Budach W, Zietman A, Freeman J, Gioioso D, Ruka W, Suit HD (1993). Quantitative comparison between the transplantability of human and murine tumors into the subcutaneous tissue of NCr/Sed-nu/nu nude and severe combined immunodeficient mice. Cancer Res.

[165] Budhu S, Wolchok J, Merghoub T (2014). The importance of animal models in tumor immunity and immunotherapy. Curr Opin Genet Dev.

[166] An Z, Jiang P, Wang X, Moossa AR, Hoffman RM (1999). Development of a high metastatic orthotopic model of human renal cell carcinoma in nude mice: benefits of fragment implantation compared to cell-suspension injection. Clin Exp Metastasis.

[167] Naito S, von Eschenbach AC, Giavazzi R, Fidler IJ (1986). Growth and metastasis of tumor cells isolated from a human renal cell carcinoma implanted into different organs of nude mice. Cancer Res.

[168] Ruggeri BA, Camp F, Miknyoczki S (2014). Animal models of disease: pre-clinical animal models of cancer and their applications and utility in drug discovery. Biochem Pharmacol.

[169] Chapman DW, Jans HS, Ma I, Mercer JR, Wiebe LI, Wuest M, Moore RB (2014). Detecting functional changes with [(18)F]FAZA in a renal cell carcinoma mouse model following sunitinib therapy. EJNMMI Res.

[170] Dos Santos C, Tijeras-Raballand A, Serova M, Sebbagh S, Slimane K, Faivre S, de Gramont A, Raymond E (2015). Effects of preset sequential administrations of sunitinib and everolimus on tumour differentiation in Caki-1 renal cell carcinoma. Br J Cancer.

[171] Joshi S, Singh AR, Durden DL (2015). Pan-PI-3 kinase inhibitor SF1126 shows antitumor and antiangiogenic activity in renal cell carcinoma. Cancer Chemother Pharmacol.

[172] Wu P, Zhang N, Wang X, Zhang C, Li T, Ning X, Gong K. The erythropoietin/erythropoietin receptor signaling pathway promotes growth and invasion abilities in human renal carcinoma cells. PLoS One. 2012;7(9):e45122. doi:10.1371/journal.pone.0045122. 10.1371/journal.pone.0045122 PMC3445554 23028796

[173] Valta MP1, Zhao H, Ingels A, Thong AE, Nolley R, Saar M, Peehl DM. Development of a realistic in vivo bone metastasis model of human renal cell carcinoma. Clin Exp Metastasis 2014;31(5):573-84. doi:10.1007/s10585-014-9651-8. 10.1007/s10585-014-9651-8 PMC4351963 24715498

[174] Beniers AJ, Peelen WP, Schaafsma HE, Beck JL, Ramaekers FC, Debruyne FM, Schalken JA (1992). Establishment and characterization of five new human renal tumor xenografts. Am J Pathol.

[175] Sharkey FE, Fogh J (1979). Metastasis of human tumors in athymic nude mice. Int J Cancer.

[176] Garofalo A, Chirivi RG, Scanziani E, Mayo JG, Vecchi A, Giavazzi R (1993). Comparative study on the metastatic behavior of human tumors in nude, beige/nude/xid and severe combined immunodeficient mice. Invasion Metastasis.

[177] Kobayashi M, Morita T, Chun NA, Matsui A, Takahashi M, Murakami T (2012). Effect of host immunity on metastatic potential in renal cell carcinoma: the assessment of optimal in vivo models to study metastatic behavior of renal cancer cells. Tumour Biol.

[178] Bear A, Clayman RV, Elbers J, Limas C, Wang N, Stone K, Gebhard R, Prigge W, Palmer J (1987). Characterization of two human cell lines (TK-10, TK-164) of renal cell cancer. Cancer Res.

[179] Grossman HB, Wedemeyer G, Ren LQ (1985). Human renal carcinoma: characterization of five new cell lines. J Surg Oncol.

[180] van Moorselaar RJA, Schalken JA, Oosterhof GON, Debruyne FMJ (1991). Use of animal models in diagnosis and treatment of renal cell carcinoma. World J Urol.

[181] Pan C, Kumar C, Bohl S, Klingmueller U, Mann M (2009). Comparative proteomic phenotyping of cell lines and primary cells to assess preservation of cell type-specific functions. Mol Cell Proteomics.

[182] Burdall SE, Hanby AM, Lansdown MR, Speirs V (2003). Breast cancer cell lines: friend or foe?. Breast Cancer Res.

[183] Hughes P, Marshall D, Reid Y, Parkes H, Gelber C (2007). The costs of using unauthenticated, over-passaged cell lines: how much more data do we need?. BioTechniques.

[184] Ertel A, Verghese A, Byers SW, Ochs M, Tozeren A (2006). Pathway-specific differences between tumor cell lines and normal and tumor tissue cells. Mol Cancer.

[185] Park JG, Ku JL, Park SY (2004). Isolation and culture of renal cancer cell lines. Methods Mol Med.

[186] Batchelder CA, Martinez ML, Duru N, Meyers FJ, Tarantal AF (2015). Three Dimensional Culture of Human Renal Cell Carcinoma Organoids. PLoS One.

[187] Mesoudi A (2011). Variable cultural acquisition costs constrain cumulative cultural evolution. PLoS One.

[188] Steffens S, Janssen M, Roos FC, Becker F, Schumacher S, Seidel C, Wegener G, Thuroff JW, Hofmann R, Stockle M (2012). Incidence and long-term prognosis of papillary compared to clear cell renal cell carcinoma--a multicentre study. Eur J Cancer.

[189] Thoma CR, Zimmermann M, Agarkova I, Kelm JM, Krek W (2014). 3D cell culture systems modeling tumor growth determinants in cancer target discovery. Adv Drug Deliv Rev.

[190] Bielecka ZF, Maliszewska-Olejniczak K, Safir IJ, Szczylik C, Czarnecka AM. Three-dimensional cell culture model utilization in cancer stem cell research. Biol Rev Camb Philos Soc. 2016. doi: 10.1111/brv.12293 10.1111/brv.12293 27545872

[191] Pickl M, Ries CH (2009). Comparison of 3D and 2D tumor models reveals enhanced HER2 activation in 3D associated with an increased response to trastuzumab. Oncogene.

[192] Krausz E, de Hoogt R, Gustin E, Cornelissen F, Grand-Perret T, Janssen L, Vloemans N, Wuyts D, Frans S, Axel A (2013). Translation of a tumor microenvironment mimicking 3D tumor growth co-culture assay platform to high-content screening. J Biomol Screen.

[193] Geraghty RJ, Capes-Davis A, Davis JM, Downward J, Freshney RI, Knezevic I, Lovell-Badge R, Masters JR, Meredith J, Stacey GN (2014). Guidelines for the use of cell lines in biomedical research. Br J Cancer.

