# Supplementary material for: Choosing the right cell line for renal cell cancer research
Source: Mol Cancer. 2016 Dec 19;15:83. doi: 10.1186/s12943-016-0565-8 (PMC5168717; doi:10.1186/s12943-016-0565-8)
Supplement: Additional file 1: — Characteristics of renal cell cancer cell lines including their origin, histology, culture conditions, and molecular characteristics. (DOCX 355 kb) [file 12943_2016_565_MOESM1_ESM.docx]

*wt*- wild-type gene, *m*- mutated, *p*- polymorphism, *nd*- no data on gene modifications, t- mRNA (transcript) detected, + protein expression, - no protein expression, C- changed protein structure/ size, ND- no data on protein, OP- original patient, SP and NSP- side population and non-side populations, * clear cell according to describing authors; before 1995, # inconsistent data

| **Cell line/ provider** | **Type/ source** | **Culture conditions** | **Molecular characteristics** | **Histological type** | **Other features** | **Databases/ web tools:** |
| --- | --- | --- | --- | --- | --- | --- |
| **769-P**  ATCC  CLSgmbh | Primary  1970-75  [1, 2] | RPMI, 2 mM Glutamine,1 mM sodium pyruvate, 10% FBS | VHL *m* -/+#  HIF1 *nd* -/+#  HIF2 *nd* t -/+#  c-MET *nd* +  VHL (codon 2, 180 - point mutations, Pro2Ser, Ile180Asn) and VHL promoter hypermethylation-no VHL mRNA, no HIF-1α  [3-5] | OP- unknown; RCC  Clear cell by *vhl* status  [1, 2] | TICs  No lung metastases, tumours by SP and NSP or no tumours in xenografts #  High VEGF  Genes mutated: 74 in CCLE (incl. CXCL12, MAPK kinases, Ncam, PDGFR, VEGFC, VHL), 317 in COSMIC (incl. ABC, MAPK kinases, PDGFR, VHL)  Mutated BAP1  [4, 6-11] | COSMIC:  [Mutations](http://amp.pharm.mssm.edu/Harmonizome/gene_set/769-P/COSMIC+Cell+Line+Gene+Mutation+Profiles)  [CNV](http://amp.pharm.mssm.edu/Harmonizome/gene_set/769-P/COSMIC+Cell+Line+Gene+CNV+Profiles)  [Expression](http://amp.pharm.mssm.edu/Harmonizome/gene_set/769-P/Klijn+et+al.%2C+Nat.+Biotechnol.%2C+2015+Cell+Line+Gene+Expression+Profiles)  CCLE:  [Mutations](http://amp.pharm.mssm.edu/Harmonizome/gene_set/769P/CCLE+Cell+Line+Gene+Mutation+Profiles)  [CNV](http://amp.pharm.mssm.edu/Harmonizome/gene_set/769P/CCLE+Cell+Line+Gene+CNV+Profiles)  [Expression](http://amp.pharm.mssm.edu/Harmonizome/gene_set/769P/CCLE+Cell+Line+Gene+Expression+Profiles)  [Drug sensitivity](http://www.cancerrxgene.org/translation/CellLine/910922) |
| **786-O**  ATCC  CLSgmbh | Primary  1970-75  [1, 2] | RPMI, 2 mM Glutamine,1 mM sodium pyruvate, 10% FBS | VHL *m* t -/+#  HIF1 *m* t -  HIF2 *nd* +  c-MET *nd* +  non-functional VHL (codon104 -1 base deletion), no HIF-1α , high HIF-2α  [3-5, 12-19] | OP- unknown; RCC  Clear cell by *vhl* status  CD10 positive  [1, 2, 20] | Agar colonies  TICs  Tumorigenic in nude mice; tumours by SP and lung metastases in xenografts  Fuhrman 4 ccRCC in mice  High VEGF  Cell line transfected with Luciferase available  Genes mutated: 90 in CCLE (incl. FGFR, IL-1, MAPK kinases, PTEN, TP53, VEGFC), 344 in COSMIC (incl. ABC, FLT1, PTEN, TP53, VHL)  [3, 4, 7, 11, 21-28] | COSMIC:  [Mutations](http://amp.pharm.mssm.edu/Harmonizome/gene_set/786-0/COSMIC+Cell+Line+Gene+Mutation+Profiles)  [CNV](http://amp.pharm.mssm.edu/Harmonizome/gene_set/786-0/COSMIC+Cell+Line+Gene+CNV+Profiles)  [Expression](http://amp.pharm.mssm.edu/Harmonizome/gene_set/786-O/Klijn+et+al.%2C+Nat.+Biotechnol.%2C+2015+Cell+Line+Gene+Expression+Profiles)  CCLE:  [Mutations](http://amp.pharm.mssm.edu/Harmonizome/gene_set/786O/CCLE+Cell+Line+Gene+Mutation+Profiles)  [CNV](http://amp.pharm.mssm.edu/Harmonizome/gene_set/786O/CCLE+Cell+Line+Gene+CNV+Profiles)  [Expression](http://amp.pharm.mssm.edu/Harmonizome/gene_set/786O/CCLE+Cell+Line+Gene+Expression+Profiles)  [Gene KO effects](http://amp.pharm.mssm.edu/Harmonizome/gene_set/786O/Achilles+Cell+Line+Gene+Essentiality+Profiles)  [Drug sensitivity](http://www.cancerrxgene.org/translation/CellLine/905947) |
| **A-498**  ATCC  CLSgmbh  DSMZ | primary  1973  [29] | EMEM, 2 mM Glutamine, 1% Non-Essential Amino Acids,1 mM sodium pyruvate, 10% FBS | VHL *m/wt#* t -  HIF1 *nd* t C  HIF2 *nd* t +  c-MET *nd* +  codones 142-3 - 4 bases deletion), no HIF-1α mRNA, high HIF-2α, SETD2 mutation,  [3, 5, 14, 18, 30, 31] | OP- kidney carcinoma  Moderately  differentiated clear cell* in xenografts  Clear cell by *vhl* status  Papillary# in xenografts and by chromosome analysis  [32, 33] | Tumorigenic in nude mice (sc and iv)  Wt p53#  TICs  High VEGF  Genes mutated: 86 in CCLE (incl. CXCL12, EGF, FGFR, MAPK kinases, Ncam, PIK3, TP53, VHL), 282 in COSMIC (incl. PIK3, VHL)  [3, 34-40] | COSMIC:  [Mutations](http://amp.pharm.mssm.edu/Harmonizome/gene_set/A498/COSMIC+Cell+Line+Gene+Mutation+Profiles)  [CNV](http://amp.pharm.mssm.edu/Harmonizome/gene_set/A498/COSMIC+Cell+Line+Gene+CNV+Profiles)  [Expression](http://amp.pharm.mssm.edu/Harmonizome/gene_set/A-498/Klijn+et+al.%2C+Nat.+Biotechnol.%2C+2015+Cell+Line+Gene+Expression+Profiles)  CCLE:  [Mutations](http://amp.pharm.mssm.edu/Harmonizome/gene_set/A498/CCLE+Cell+Line+Gene+Mutation+Profiles)  [CNV](http://amp.pharm.mssm.edu/Harmonizome/gene_set/A498/CCLE+Cell+Line+Gene+CNV+Profiles)  [Expression](http://amp.pharm.mssm.edu/Harmonizome/gene_set/A498/CCLE+Cell+Line+Gene+Expression+Profiles)  [Drug sensitivity](http://www.cancerrxgene.org/translation/CellLine/905948) |
| **A-704**  ATCC | primary  1973  [29] | EMEM, 2 mM Glutamine, 1% Non-Essential Amino Acids, 1 mM sodium pyruvate, 10% FBS | VHL *m* -  HIF1 *nd* +  HIF2 *nd* +  c-MET *nd* ND  [41, 42] | OP- kidney carcinoma  Clear cell by *vhl* status  [29] | Not tumorigenic in nude mice  Genes mutated: 477 in COSMIC (incl. FGF, FGFR, MAPK kinases, TLR5, VHL), 105 in CCLE (incl. BAX, EGF, HGF, Ncam, PDGFR, PIK3, TLR3, TNFR, VEGF, VHL)  Mutated PBRM1  [43, 44] | COSMIC:  [Mutations](http://amp.pharm.mssm.edu/Harmonizome/gene_set/A704/COSMIC+Cell+Line+Gene+Mutation+Profiles)  [CNV](http://amp.pharm.mssm.edu/Harmonizome/gene_set/A704/COSMIC+Cell+Line+Gene+CNV+Profiles)  CCLE:  [Mutations](http://amp.pharm.mssm.edu/Harmonizome/gene_set/A704/CCLE+Cell+Line+Gene+Mutation+Profiles)  [CNV](http://amp.pharm.mssm.edu/Harmonizome/gene_set/A704/CCLE+Cell+Line+Gene+CNV+Profiles)  [Expression](http://amp.pharm.mssm.edu/Harmonizome/gene_set/A704/CCLE+Cell+Line+Gene+Expression+Profiles)  [Drug sensitivity](http://www.cancerrxgene.org/translation/CellLine/910920) |
| **ACHN**  ATCC  CLSgmbh  Sigma  PHE | Metastatic (pleural effusion)  1979  [45] | 1.EMEM, 2 mM Glutamine, 1% non-essential amino acids, 1 mM sodium pyruvate, 10% FBS  2. DMEM, 10% FBS, 2 mM Glutamine | VHL *wt* +  HIF1 *nd* +  HIF2 *nd* +  c-MET *p* +  [3-5, 12, 18, 30, 46, 47] | OP- unknown; RCC  Clear cell by gene expression analysis  Papillary# by copy number alteration and chromosome analysis  Poorly differentiated clear cell*in xenografts  [33, 45, 48-50] | Growth inhibition by IFN  Agar colonies  Sphere formation  TICs  Wt p53  Tumorigenic in nude mice  Tumours by SP in xenografts  Genes mutated: 168 in COSMIC (incl. ABCA genes, MAPK kinases, PBMR1#, TLR2), 62 in CCLE (incl. Ncam, MAPK kinases, PIK3, VEGFC). PBMR1 wildtype/m#  [34, 36, 51-61] | COSMIC:  [Mutations](http://amp.pharm.mssm.edu/Harmonizome/gene_set/ACHN/COSMIC+Cell+Line+Gene+Mutation+Profiles)  [CNV](http://amp.pharm.mssm.edu/Harmonizome/gene_set/ACHN/COSMIC+Cell+Line+Gene+CNV+Profiles)  [Expression](http://amp.pharm.mssm.edu/Harmonizome/gene_set/ACHN/Klijn+et+al.%2C+Nat.+Biotechnol.%2C+2015+Cell+Line+Gene+Expression+Profiles)  CCLE:  [Mutations](http://amp.pharm.mssm.edu/Harmonizome/gene_set/ACHN/CCLE+Cell+Line+Gene+Mutation+Profiles)  [CNV](http://amp.pharm.mssm.edu/Harmonizome/gene_set/ACHN/CCLE+Cell+Line+Gene+CNV+Profiles)  [Expression](http://amp.pharm.mssm.edu/Harmonizome/gene_set/ACHN/CCLE+Cell+Line+Gene+Expression+Profiles)  [Gene KO effects](http://amp.pharm.mssm.edu/Harmonizome/gene_set/ACHN/Achilles+Cell+Line+Gene+Essentiality+Profiles)  [Drug sensitivity](http://www.cancerrxgene.org/translation/CellLine/905950) |
| **Caki-1**  ATCC  CLSgmbh  DSMZ  MSKCC  JCRB | Metastatic (skin)  1975  [62] | 1. McCoy's 5a, 10% FBS, 2 mM Glutamine  2. EMEM, 2 mM Glutamine, 1% non-essential amino acids | VHL *wt* +  HIF1 *m* +  HIF2 *nd* +  c-MET *m* +  [3, 5, 18, 19, 63-65] | OP- adenocarcinoma  Poorly differentiated clear cell*or clear cell in xenografts  [33, 53, 62] | Sphere formation  Wt p53  Tumorigenic, lung metastases  Tumours with sarcomatoid changes by SP in xenografts  High VEGF  Cell line transfected with Luciferase available  Genes mutated: 76 in CCLE (incl. HIF1A, MAPK kinases, MET, MMP9, Ncam, VEGFC), 336 in COSMIC (incl. ABC, FGFR, HLA, IL-10R, MAPK kinases, MET, NES, PIUK3, TRAF)  [4, 23, 36, 65-68] | COSMIC:  [Mutations](http://amp.pharm.mssm.edu/Harmonizome/gene_set/CAKI-1/COSMIC+Cell+Line+Gene+Mutation+Profiles)  [CNV](http://amp.pharm.mssm.edu/Harmonizome/gene_set/CAKI-1/COSMIC+Cell+Line+Gene+CNV+Profiles)  [Expression](http://amp.pharm.mssm.edu/Harmonizome/gene_set/CAKI-1/Klijn+et+al.%2C+Nat.+Biotechnol.%2C+2015+Cell+Line+Gene+Expression+Profiles)  CCLE:  [Mutations](http://amp.pharm.mssm.edu/Harmonizome/gene_set/CAKI1/CCLE+Cell+Line+Gene+Mutation+Profiles)  [CNV](http://amp.pharm.mssm.edu/Harmonizome/gene_set/CAKI1/CCLE+Cell+Line+Gene+CNV+Profiles)  [Expression](http://amp.pharm.mssm.edu/Harmonizome/gene_set/CAKI1/CCLE+Cell+Line+Gene+Expression+Profiles)  [Drug sensitivity](http://www.cancerrxgene.org/translation/CellLine/905963) |
| **Caki-2**  DSMZ  Sigma  MSKCC  PHE | Primary  1975  [62] | 1. McCoy's 5a, 10% FBS, 2 mM Glutamine  2. EMEM, 2 mMGlutamine, 1% non-essential amino acids | VHL *wt/m#* -  HIF1 *nd* +  HIF2 *nd* +  c-MET *nd* +  [3, 5, 14, 18, 30, 69] | OP- adenocarcinoma  Papillary# by MET expression and in xenografts  Papillary in xenografts  [33, 48, 62, 69, 70] | Wt p53  TICs  Tumorigenic in nude mice  Tumours by SP  Genes mutated: 80 in CCLE (incl. ABC, EGF, FGFR, MAPK kinases, Ncam, PDGFR, VEGF, VHL#)  Mutated PBRM1  [36, 57, 61, 71] | COSMIC:  [Expression](http://amp.pharm.mssm.edu/Harmonizome/gene_set/CAKI-2/Klijn+et+al.%2C+Nat.+Biotechnol.%2C+2015+Cell+Line+Gene+Expression+Profiles)  CCLE:  [Mutations](http://amp.pharm.mssm.edu/Harmonizome/gene_set/CAKI2/CCLE+Cell+Line+Gene+Mutation+Profiles)  [CNV](http://amp.pharm.mssm.edu/Harmonizome/gene_set/CAKI2/CCLE+Cell+Line+Gene+CNV+Profiles)  [Expression](http://amp.pharm.mssm.edu/Harmonizome/gene_set/CAKI2/CCLE+Cell+Line+Gene+Expression+Profiles) |
| **EKCSC**  Promab | Primary kidney cancer | Dedicated medium from the provider | Donor dependant  VHL *nd* ND  HIF1 *nd* ND  HIF2 *nd* ND  c-MET *nd* ND | OP- no data | Enriched Kidney Cancer Stem Cell From Primary Cancer Tissue |  |
| **HEK293**  DSMZ  RBRC  Sigma  CLSgmbh  Gene And Cell Technologies | Foetal kidney  1973  [72] | 1. DMEM (high glucose), 10% FBS, 2 mM Glutamine  2. MEM, 10% FBS, 0.1 mM NEAA | VHL *wt* +  HIF1 *nd* +  HIF2 *nd* +  c-MET *nd* +  [17, 73, 74] | Embryonic kidney cells  Transformed with adenovirus  [72] | Transcriptome similar to adrenal gland, with neural properties  Tumorigenic in xenografts (high passage)  VEGF production  [75-77] | [HEK293genome](http://hek293genome.org/v2/index.php)  Gene expression:  [BioGPS](http://amp.pharm.mssm.edu/Harmonizome/gene_set/HEK293/BioGPS+Cell+Line+Gene+Expression+Profiles)  [HPA](http://amp.pharm.mssm.edu/Harmonizome/gene_set/hek293/HPA+Cell+Line+Gene+Expression+Profiles)  [78] |
| **HK2**  ATCC | Healthy kidney, cortex/proximal tubule  [79] | Keratinocyte Serum Free Medium (supplemented with L-glutamine, human Epidermal Growth Factor 1-53 and Bovine Pituitary Extract) | VHL *nd* +  HIF1 *nd* +  HIF2 *nd* +  c-MET *nd* ND  [64] | Normal renal cells, immortalised; human papillomavirus 16 (HPV-16) transformed  [79] |  | CCLE:  [CNV](http://amp.pharm.mssm.edu/Harmonizome/gene_set/HK2/CCLE+Cell+Line+Gene+CNV+Profiles)  [Expression](http://amp.pharm.mssm.edu/Harmonizome/gene_set/HK2/CCLE+Cell+Line+Gene+Expression+Profiles) |
| **HKCSCs**  Celprogen | Human Kidney Cancer Stem Cells, from single donors | Dedicated medium and flasks provided by the manufacturer | Donor dependant  VHL *nd* ND  HIF1 *nd* ND  HIF2 *nd* ND  c-MET *nd* ND | Unknown histology; papillary (unpublished, personal communication) | Positive for: CD133, CD44, SSEA3/4, Oct4, Alkaline Phosphatase, Aldehyde Dehydrogenase, Telomerase, Sox 2 (unpublished, providers information)  Tumorigenic (unpublished, providers information) |  |
| **HRC-31**  Fox Chase Cancer Centre |  | RPMI 1640, 2 mM Glutamine, 1 mM sodium pyruvate, 10% FBS | VHL *wt* +  HIF1 *nd* -  HIF2 *nd* +  c-MET *nd* ND  [80] | Clear cell by copy number alteration and IHC staining  [80] |  |  |
| **HRC-45**  Fox Chase Cancer Centre |  | RPMI 1640 medium, 2 mM Glutamine, 1 mM sodium pyruvate, 10% FBS | VHL *nd* +  HIF1 *nd* +  HIF2 *nd* ND  c-MET *nd* ND  [80] | Clear cell by copy number alteration and IHC staining  [80] |  |  |
| **HRC-63**  Fox Chase Cancer Centre |  |  | VHL *nd* -  HIF1 *nd* +  HIF2 *nd* ND  c-MET *nd* ND  [80] | Clear cell by copy number alteration and IHC staining  [80] |  |  |
| **HRCE**  Lonza  ScienCell  PromoCell | Renal Cortical Epi Cells | 1. Dedicated medium (low serum, supplemented with human transferrin hydrocortisone, insulin, triiodothyronine, epinephrine, EGF)  2. RPMI 1640: Click's Medium (1:1) | Donor dependant  VHL *nd* ND  HIF1 *nd* ND  HIF2 *nd* ND  c-MET *nd* ND  [81] | Healthy, primary renal cortical cells |  |  |
| **HRE**  Lonza  ScienCell  PromoCell  Applied StemCell | Renal Epithelial Cells | Dedicated medium (low serum) | Donor dependant  VHL *nd* ND  HIF1 *nd* ND  HIF2 *nd* ND  c-MET *nd* ND | Healthy, primary renal epithelial cells |  |  |
| **KH39** | Primary  1981  [82] | Ham's F12 with 20% FCS | VHL *nd* ND  HIF1 *nd* ND  HIF2 *nd* ND  c-MET *nd* ND |  | Tumorigenic in nude mice  Hemizygotic Ch3  Wt p53  [36, 83, 84] |  |
| **KMRC-2**  **JCRB Cell Bank** | Primary  1999  [18] | DMEM, 10% FBS, 2 mM Glutamine | VHL *m* -  HIF1 *nd* +  HIF2 *nd* ND  c-MET *nd* ND  [18] | OP- no data  Clear cell by *vhl* status | Genes mutated: 85 in CCLE (incl. ABC, FGFGR, MAPK kinases, Ncam, TGF, VEGFC)  [85] | CCLE:  [Mutations](http://amp.pharm.mssm.edu/Harmonizome/gene_set/KMRC2/CCLE+Cell+Line+Gene+Mutation+Profiles)  [CNV](http://amp.pharm.mssm.edu/Harmonizome/gene_set/KMRC2/CCLE+Cell+Line+Gene+CNV+Profiles)  [Expression](http://amp.pharm.mssm.edu/Harmonizome/gene_set/KMRC2/CCLE+Cell+Line+Gene+Expression+Profiles) |
| **KRC/Y** | Primary  1987  [86] | 1. RPMI, 10% FBS, 2 mM Glutamine  2. Iscove's Modified Dulbecco's Medium, 10% FBS, 2 mM Glutamine, 1 mM sodium pyruvate, 0.4 µg/ml hydrocortisone | VHL *wt* +  HIF1*nd* t ND  HIF2 *nd* ND  c-MET *nd* ND  [30] | OP- tumour cystic and necrotic, yellow, fibrous capsule, both clear* and granular cells  Clear* and granular cells in xenografts  [86] | SP equally tumorigenic as NSP  Sphere formation  Mutated and overexpressed p53  Terminal deletion of Ch3  [36, 52, 84] |  |
| **NHMC**  Lonza | Mesangial Cells | Dedicated medium provided by the manufacturer | Donor dependant  VHL *nd* ND  HIF1 *nd* ND  HIF2 *nd* ND  c-MET *nd* ND |  |  |  |
| **OS-RC-2**  RBRC | Primary  1984  [87] | 1. RPMI 1640, 10% FBS  2. DMEM/F12, 10% FBS | VHL *m* +  HIFa *nd* ND  HIF2 *nd* ND  c-MET *nd* ND  [88, 89] | Clear cell metastases in xenografts  Clear cell by *vhl* status  [90] | Tumorigenic in nude mice  Genes mutted: 385 in COSMIC (incl. ABC, HLA, PBRM1, PTEN, VHL, WT1)  [90-92] | COSMIC:  [Mutations](http://amp.pharm.mssm.edu/Harmonizome/gene_set/OS-RC-2/COSMIC+Cell+Line+Gene+Mutation+Profiles)  [CNV](http://amp.pharm.mssm.edu/Harmonizome/gene_set/OS-RC-2/COSMIC+Cell+Line+Gene+CNV+Profiles)  CCLE:  [Mutations](http://amp.pharm.mssm.edu/Harmonizome/gene_set/OSRC2/CCLE+Cell+Line+Gene+Mutation+Profiles)  [CNV](http://amp.pharm.mssm.edu/Harmonizome/gene_set/OSRC2/CCLE+Cell+Line+Gene+CNV+Profiles)  [Expression](http://amp.pharm.mssm.edu/Harmonizome/gene_set/OSRC2/CCLE+Cell+Line+Gene+Expression+Profiles)  [Drug sensitivity](http://www.cancerrxgene.org/translation/CellLine/909250) |
| **PSC-400-010**  ATCC | Primary Renal Proximal Tubule Epithelial Cells | Dedicated medium | VHL *nd* ND  HIF1 *nd* ND  HIF2 *nd* ND  c-MET *nd* ND | Healthy, primary renal epithelial cells |  |  |
| **RCC 10** | Primary ccRCC (VHL Defecting) | 1. RPMI 1640, 10%FBS  2. DMEM, 10% FBS, 2 mM Glutamine | VHL *m* -  HIF1 *nd* +  HIF2 *nd* +  c-MET *nd* ND  [93-97] | OP- clear cell  Clear cell by *vhl* status  [97] | Tumorigenic in nude mice; non-tumorigenic after VHL reconstitution  [97] |  |
| **RCC6** | Primary  2002  [98, 99] | DMEM, 10% FCS, 1% ultroserG | VHL *m* t -/+#  HIF1 *nd* +  HIF2 *nd* +  c-MET *nd* ND  [100] | OP- clear cell, with metastases  [99, 101] |  |  |
| **RCC6** | Primary  1994  [102] | DMEM, 10% FCS | VHL *m* -  HIF1 *nd* t ND  HIF2 *nd* ND  c-MET *nd* ND  [102] | OP- papillary/ clear cell#  [97, 102] |  |  |
| **RCC4**  Sigma  PHE | No data | DMEM, 10% FCS | VHL *m* -  HIF1 *nd* +  HIF2 *nd* +  c-MET *nd* ND  [93, 94, 103] | OP- no data  CD10 positive  Clear cell by *vhl* status  [20] | Stable *vhl* transfected cell line commercially available  Tumorigenic in nude mice (providers information)  [104] | CCLE:  [Expression](http://amp.pharm.mssm.edu/Harmonizome/gene_set/RCC4/CCLE+Cell+Line+Gene+Expression+Profiles) |
| **RCC BA85#21**  PHE | Primary  2004  [105] | RPMI 1640, 2 mM Glutamine, 20% FBS | VHL *nd* ND  HIF1 *nd* ND  HIF2 *nd* ND  c-MET *nd* ND | OP- clear cells with alveolar/ tubular arrangement  [105] | Induction of antigen specific immunogenic response- patented  [105] |  |
| **RCC-ER**  **(KTCTL-13)**  CLSgmbh  CosmoBio  XZell | Primary  Before 1993 | RPMI 1640, 2 mM Glutamine, 10% FBS | VHL *m* ND  HIF1 *nd* ND  HIF2 *nd* ND  c-MET *nd* ND  [31, 106] | OP- 57-years-old-male, kidney clear cell carcinoma pT3a, N1, Mx/GIII (providers information)  Clear cell by *vhl* status  [31] | Tumorigenic in nude mice (providers information)  Genes mutated: 288 in COSMIC (incl. ABC, MTOR, PBRM1, TLR6, VHL)  Mutated SETD2  [106, 107] | COSMIC:  [Mutations](http://amp.pharm.mssm.edu/Harmonizome/gene_set/RCC-ER/COSMIC+Cell+Line+Gene+Mutation+Profiles)  [CNV](http://amp.pharm.mssm.edu/Harmonizome/gene_set/RCC-ER/COSMIC+Cell+Line+Gene+CNV+Profiles)  [Drug sensitivity](http://www.cancerrxgene.org/translation/CellLine/1524417) |
| **RCC-EW**  **(KTCLT-2)**  CLSgmbh  XZell | Primary  Before 1993 | RPMI 1640, 2 mM Glutamine, 10% FBS | VHL *m* ND  HIF1 *nd* ND  HIF2 *nd* ND  c-MET *nd* ND  [31] | OP- kidney Adenocarcinoma pT3a, N1, Mx/GIII (providers information)  Clear cell by *vhl* status  [31] | Tumorigenic in nude mice (providers information) |  |
| **RCC-FG1**  **(KTCTL-26)**  CLSgmbh  XZell | Primary  Before 1993  [108] | RPMI 1640, 2 mM Glutamine, 10% FBS | VHL *m* ND  HIF1 *nd* ND  HIF2 *nd* ND  c-MET *nd* ND  [31] | OP- clear cell carcinoma pT2a, M1/GII (providers information)  Clear cell by *vhl* status  [31] | Ch3 loss  Tumorigenic in nude mice (providers information)  [108-110] |  |
| **RPTEC**  ATCC  Lonza  Evercyte | Renal Proximal Tubule Cells | Dedicated medium | Donor dependant  VHL *wt* +  HIF1 *wt* +  HIF2 *wt* +  c-MET *wt* +  [4, 111] | Healthy, primary renal epithelial cells | Immortalized cell line available  [112, 113] |  |
| **SKRC39**  MSKCC | Metastatic (soft tissue)  1972-87  [114] | 1. EMEM, 7,5% FBS, 1% NEAA, 1% PS, 2 mM Glutamine  2. RPMI, 10% FCS | VHL *wt* +  HIF1 *nd* ND  HIF2 *nd* ND  C-MET *nd* +  [30] | OP- no data  Papillary by LRRK2 and MET expression  [69] | Tumorigenic in nude mice  Mutation of *bhd*  [114, 115] |  |
| **SKRC1**  MSKCC | Primary  1972-87  [114] | 1. EMEM, 7,5% FBS, 1% NEAA, 1% PS, 2 mM Glutamine  2. RPMI, 10% FCS | VHL *m* ND  HIF1 *nd* ND  HIF2 *nd* ND  c-MET *nd* ND  [18] | OP- no data  Clear cell by *vhl* status | Tumorigenic in nude mice  [114] |  |
| **SKRC7**  MSKCC | Primary  1972-87  [114] | 1. EMEM, 7,5% FBS, 1% NEAA, 1% PS, 2 mM Glutamine  2. RPMI, 10% FCS | VHL *m* ND  HIF1 *nd* +  HIF2 *nd* +  c-MET *nd* ND  [18, 116, 117] | OP- no data  Clear cell by *vhl* status | Not tumorigenic in nude mice  [114] |  |
| **SKRC45**  MSKCC | Metastatic (adrenal)  1972-87  [114] | 1. EMEM, 7,5% FBS, 1% NEAA, 1% PS, 2 mM Glutamine  2. RPMI, 10% FCS | VHL *m* ND  HIF1 *nd* ND  HIF2 *nd* ND  c-MET *nd* ND  [30] | OP- no data  Clear cell by *vhl* status | Tumorigenic in nude mice  [114] |  |
| **SKRC48**  MSKCC | Kidney Cancer Primary  1972-87 | 1. EMEM, 7,5% FBS, 1% NEAA, 1% PS, 2 mM Glutamine  2. RPMI, 10% FCS | VHL *m* ND  HIF1 *nd* ND  HIF2 *nd* ND  c-MET *nd* ND  [30, 31] | OP- no data  Clear cell by *vhl* status | Tumorigenic in nude mice  [114] |  |
| **SMKT-R1** | Primary  1986  [118] | DMEM, 10% FCS | VHL *nd* ND  HIF1 *nd* ND  HIF2 *nd* ND  c-MET *nd* ND | OP- alveolar type, clear cell* subtype, pT2b, pV0 and grade 2  [118] | Tumorigenic in nude mice  High VEGF  [118, 119] |  |
| **SMKT-R2** | Primary; xenotransplant  1986  [118] | DMEM, 10% FCS | VHL *m* ND  HIF1 *nd* +  HIF2 *nd* +  c-MET *nd* ND  [18, 120] | OP- alveolar type and clear cell* predominant-mixed subtype, pT3, pVla and grade 2  Clear cell by *vhl* status  [118] | Tumorigenic in nude mice  High VEGF  [118, 119] |  |
| **SMKT-R3** | Primary; xenotransplant  1987  [118] | DMEM, 10% FCS | VHL *m* ND  HIF1 *nd* +  HIF2 *nd* +  c-MET *nd* +  [18, 120, 121] | OP- papillary type and granular cell subtype, pT3, pV1 a and grade 2> 3  Clear cell by *vhl* status  [118] | Tumorigenic in nude mice  [118] |  |
| **SN12-C** | Primary  1986  [82] | DMEM, 10% FCS | VHL *wt* +  HIF1 *nd* +  HIF2 *nd* +  c-MET *nd* ND  [93, 122] | Mixed granular/ clear cell* morphology  Clear cell by chromosome analysis  [123] | Tumorigenic in nude mice, liver metastasis  Genes mutated: 815 in COSMIC (incl. ABC, E-CDH, EGFR, PGF, TLR5, TP53, KDM6A)  [82, 124, 125] | COSMIC:  [Mutations](http://amp.pharm.mssm.edu/Harmonizome/gene_set/SN12C/COSMIC+Cell+Line+Gene+Mutation+Profiles)  [CNV](http://amp.pharm.mssm.edu/Harmonizome/gene_set/SN12C/COSMIC+Cell+Line+Gene+CNV+Profiles)  [Drug sensitivity](http://www.cancerrxgene.org/translation/CellLine/905979) |
| **SNU 1272**  KCLB | Primary  1994  [126] | RPMI 1640, 2 mM Glutamine, 10% FBS | VHL *m* ND  HIF1 *nd* ND  HIF2 *nd* ND  c-MET *nd* ND  [126] | OP- clear cell, alveolar, polygonal cells. Tumour with necrosis, no metastases, grade 2.  Clear cell by *vhl* status  [126] | Genes mutated: 86 in CCLE (incl. HIF1A, MAPK kinases, Ncam, PIK3, TGF, VEGFC, VHL)  [127] | CCLE:  [Mutations](http://amp.pharm.mssm.edu/Harmonizome/gene_set/SNU1272/CCLE+Cell+Line+Gene+Mutation+Profiles)  [CNV](http://amp.pharm.mssm.edu/Harmonizome/gene_set/SNU1272/CCLE+Cell+Line+Gene+CNV+Profiles)  [Expression](http://amp.pharm.mssm.edu/Harmonizome/gene_set/SNU1272/CCLE+Cell+Line+Gene+Expression+Profiles) |
| **SNU 228**  KCLB | Primary  1989  [126] | RPMI 1640, 2 mM Glutamine, 10% FBS | VHL *wt* ND  HIF1 *nd* ND  HIF2 *nd* ND  c-MET *nd* ND | OP- clear cell/ unknown  [126] |  |  |
| **SNU 267**  KCLB | Primary  1989  [126] | RPMI 1640, 2 mM Glutamine, 10% FBS | VHL *wt* ND  HIF1 *nd* ND  HIF2 *nd* ND  c-MET *nd* ND  [126] | OP- clear cell, alveolar, tubular, spindle cells. Tumour without necrosis, no metastases, grade 1.  [126] | Mutated p53 (no mutation in original tumour)  [126] |  |
| **SNU 328**  KCLB | Primary  1990  [126] | RPMI 1640, 2 mM Glutamine, 10% FBS | VHL *wt* ND  HIF1 *nd* ND  HIF2 *nd* ND  c-MET *nd* ND  [126] | OP- clear cell, alveolar, solid, round cells. Tumour with necrosis, no metastases, grade 2.  [126] |  |  |
| **SNU 333**  KCLB | Primary  1990  [126] | RPMI 1640, 2 mM Glutamine, 10% FBS | VHL *wt* ND  HIF1 *nd* ND  HIF2 *nd* ND  c-MET *nd* ND  [126] | OP- clear cell, alveolar, granular, solid, spindle cells. Tumour with necrosis, no metastases, grade 3.  [126] |  |  |
| **SNU 349**  KCLB | Primary  1990  [126] | RPMI 1640, 2 mM Glutamine, 10% FBS | VHL *m* ND  HIF1 *m* ND  HIF2 *nd* ND  c-MET *nd* ND  [126] | OP- clear cell, tubular, polygonal cells. Tumour with necrosis, no metastases, grade 1.  Clear cell by *vhl* status  [126] | Genes mutated: 171 in CCLE (incl. BAX, BRCA, FASL, HIF1A, IL2, MAPK kinases, MTOR, Ncam, PIK3, STAT1, STAT3, TNFR, VEGFC)  [128] | CCLE:  [Mutations](http://amp.pharm.mssm.edu/Harmonizome/gene_set/SNU349/CCLE+Cell+Line+Gene+Mutation+Profiles)  [CNV](http://amp.pharm.mssm.edu/Harmonizome/gene_set/SNU349/CCLE+Cell+Line+Gene+CNV+Profiles)  [Expression](http://amp.pharm.mssm.edu/Harmonizome/gene_set/SNU349/CCLE+Cell+Line+Gene+Expression+Profiles) |
| **SNU 482**  KCLB | Primary  1990  [126] | RPMI 1640, 2 mM Glutamine, 10% FBS | VHL *wt* ND  HIF1 *nd* ND  HIF2 *nd* ND  c-MET *nd* ND  [126, 128] | OP- papillary*, tubular, stellate cells. Tumour with necrosis, metastases and invasion to artery, grade 3.  [126] |  |  |
| **SW-839** | No data | RPMI 1640, 2 mM Glutamine, 10% FBS | VHL *m* ND  HIF1 *nd* t -  HIF2 *nd* +  c-MET *nd* ND  VHL codone 142-3 4 bp deletion – stop at 157  [3] | OP- no data  Clear cell by *vhl* status | High VEGF  Tumorigenic in nude mice  [3, 8, 129] |  |
| **TK 10**  National Cancer Institute,Frederick, | Primary  1980  [130] | DMEM, 10% FCS | VHL *wt +*  HIF1 *nd* +  HIF2 *nd* +  c-MET *nd* ND  [131, 132] | OP: necrotic, soft yellow tumour , Stage I or T_2_N_X_M_0_, cells large, pleomorphic, epithelial. Papillary and glandular structures; a spindle cell pattern. Nuclei large, irregular, with prominent nucleoli.  [130] | Not tumorigenic in nude mice  Agar colonies  Genes mutated: 516 in COSMIC (incl. ABC, HLA, MAPK kinases, PIK3, TLR2)  [130, 133] | COSMIC:  [Mutations](http://amp.pharm.mssm.edu/Harmonizome/gene_set/TK10/COSMIC+Cell+Line+Gene+Mutation+Profiles)  [CNV](http://amp.pharm.mssm.edu/Harmonizome/gene_set/TK10/COSMIC+Cell+Line+Gene+CNV+Profiles)  [Expression](http://amp.pharm.mssm.edu/Harmonizome/gene_set/TK-10/Klijn+et+al.%2C+Nat.+Biotechnol.%2C+2015+Cell+Line+Gene+Expression+Profiles)  [Drug sensitivity](http://www.cancerrxgene.org/translation/CellLine/905980) |
| **TK 164** | Primary  1982  [130] | DMEM, 10% FCS | VHL *nd* ND  HIF1 *nd* ND  HIF2 *nd* ND  c-MET *nd* ND | OP: yellow, nodular tumour, clear cell and granular with abundant glycogen and irregular nuclei, Stage Ilia or T3abNoMo,  [130] | Not tumorigenic in nude mice  Agar colonies  Terminal deletion of Ch3  [84, 130] |  |
| **UM-RC-2**  Sigma  PHE | Primary  1984  [134] | EMEM, 2 mM Glutamine, 1% NEAA, 10% FBS | VHL *m* -  HIF1 *m* +  HIF2 *nd* ND  c-MET *nd* ND  [15, 31] | OP- renal adenocarcinoma  Clear cell by *vhl* status  [134] | Tumorigenic in nude mice  [15, 134] | [Expression](http://amp.pharm.mssm.edu/Harmonizome/gene_set/UMRC2/CCLE+Cell+Line+Gene+Expression+Profiles) |
| **UM-RC-3**  Sigma  PHE | Primary  1984  [134] | EMEM, 2 mM Glutamine, 1% NEAA, 10% FBS | VHL *m* ND  HIF1 *nd* ND  HIF2 *nd* ND  c-MET *nd* +  [19, 31] | OP- renal adenocarcinoma  Clear cell by *vhl* status  [134] | Tumorigenic in nude mice  [134] |  |
| **UM-RC-6**  Sigma  PHE | Primary  1984  [134] | 1. DMEM, 10% FCS, 1% PS  2. EMEM, 2 mM Glutamine, 1% NEAA, 10% FBS | VHL *m* -  HIF1 *m* +  HIF2 *nd* +  c-MET *nd* +  [15, 18, 19, 31, 93, 111, 135, 136] | OP- renal adenocarcinoma  Clear cell by *vhl* status  [134] | Not tumorigenic in nude mice  Mutated BAP1  [11, 134] | [Expression](http://amp.pharm.mssm.edu/Harmonizome/gene_set/UMRC6/CCLE+Cell+Line+Gene+Expression+Profiles) |
| **UO31**  National Cancer Institute, Frederick | Primary | RPMI 1640, 2mM Glutamine, 10% FBS | VHL *wt* ND  HIF1 *nd* ND  HIF2 *nd* ND  c-MET *nd* ND  [34] | OP- no data |  | COSMIC:  [CNV](http://amp.pharm.mssm.edu/Harmonizome/gene_set/U031/COSMIC+Cell+Line+Gene+CNV+Profiles)  CCLE:  [CNV](http://amp.pharm.mssm.edu/Harmonizome/gene_set/UO31/CCLE+Cell+Line+Gene+CNV+Profiles) |
| **UOK 108**  NIH, NCI, Bethesda | Primary  1991  [137] | DMEM, 2mM Glutamine, 10% FBS | VHL *wt* ND  HIF1 *nd* ND  HIF2 *nd* ND  c-MET *nd* ND  [31, 138] | OP- clear cell*  [137, 139] | Chromosome 3 loss  Not tumorigenic in nude mice  [139] |  |
| **UOK 117**  NIH, NCI, Bethesda | Primary  1991  [137] | DMEM, 2mM Glutamine, 10% FBS | VHL *m* t ND  HIF1 *nd* ND  HIF2 *nd* ND  c-MET *nd* ND  [31, 140] | OP- clear cell*/ granular/ sarcomatoid  Clear cell by *vhl* status  [137] | Tumorigenic in nude mice  [137] |  |
| **UOK 121**  NIH, NCI, Bethesda | Primary  1991  [137] | DMEM, 2mM Glutamine, 10% FBS | VHL *wt* -  HIF1 *nd* -  HIF2 *nd* +  c-MET *nd* ND  [31, 135, 138, 140-143] | OP- granular, with metastases  [137, 139] | Methylated *vhl*  Chromosome 3 loss  Tumorigenic in nude mice  [137, 139] |  |
| **UOK 125**  NIH, NCI, Bethesda | Primary  1991  [137] | DMEM, 2mM Glutamine, 10% FBS | VHL *wt* ND  HIF1 *nd* ND  HIF2 *nd* ND  c-MET *nd* ND  [31] | OP- clear cell*  [137] | Chromosome 3 loss  Not tumorigenic in nude mouse  [137] |  |
| **UOK 127**  NIH, NCI, Bethesda | Primary  1991  [137] | DMEM, 2 mM Glutamine, 10% FBS | VHL *wt* ND  HIF1 *nd* +  HIF2 *nd* +  c-MET *nd* ND  [31, 138, 142] | OP- clear cell*/ granular/ sarcomatoid  [137] | Chromosome 3 loss  Tumorigenic in nude mice  [137] |  |
| **UOK145**  NIH, NCI, Bethesda | Primary  [144] | DMEM, 2 mM Glutamine, 10% FBS | VHL *nd* ND  HIF1 *nd* ND  HIF2 *nd* ND  c-MET *nd* ND | Papillary  [144-146] |  |  |
| **UOK171**  NIH, NCI, Bethesda | Primary  (originators [webpage](https://www.ibridgenetwork.org/#!/profiles/102/innovations/917/)) | DMEM, 2 mM Glutamine, 10% FBS | VHL *wt* ND  HIF1 *nd* ND  HIF2 *nd* ND  c-MET *nd* ND  [140, 141] | OP- clear cell (originators webpage) |  |  |
| **VMRC-RCW**  JCRB |  | RPMI 1640, 10% FBS | VHL *nd* ND  HIF1*nd* ND  HIF2*nd* ND  c-MET *nd* ND |  | Wt p53  [147] |  |
| **VMRC-RCZ**  JCRB |  | EMEM, 10% FBS | VHL *nd* ND  HIF1*nd* ND  HIF2*nd* ND  c-MET *nd* ND |  | Splicing abnormalities in p53  [147] |  |
| **WT-CLS1**  CLSgmbh | Primary  [148] | Iscove’s medium, Glutamine, supplements, 10% FBS | VHL *nd* ND  HIF1 *nd* ND  HIF2 *nd* ND  c-MET *nd* ND | **OP- Wilm'sTumor**  **Epithelial phenotype, not characteristic morphology of WT in xenografts**  [148] | Tumorigenic  [148] |  |

1. Williams RD, Elliott AY, Stein N, Fraley EE: **In vitro cultivation of human renal cell cancer. I. Establishment of cells in culture**. *In Vitro* 1976, **12**(9):623-627.

2. Williams RD, Elliott AY, Stein N, Fraley EE: **In vitro cultivation of human renal cell cancer. II. Characterization of cell lines**. *In Vitro* 1978, **14**(9):779-786.

3. Shinojima T, Oya M, Takayanagi A, Mizuno R, Shimizu N, Murai M: **Renal cancer cells lacking hypoxia inducible factor (HIF)-1alpha expression maintain vascular endothelial growth factor expression through HIF-2alpha**. *Carcinogenesis* 2007, **28**(3):529-536.

4. Miyake M, Goodison S, Lawton A, Zhang G, Gomes-Giacoia E, Rosser CJ: **Erythropoietin is a JAK2 and ERK1/2 effector that can promote renal tumor cell proliferation under hypoxic conditions**. *Journal of hematology & oncology* 2013, **6**:65.

5. Gibney GT, Aziz SA, Camp RL, Conrad P, Schwartz BE, Chen CR, Kelly WK, Kluger HM: **c-Met is a prognostic marker and potential therapeutic target in clear cell renal cell carcinoma**. *Ann Oncol* 2013, **24**(2):343-349.

6. Huang B, Huang YJ, Yao ZJ, Chen X, Guo SJ, Mao XP, Wang DH, Chen JX, Qiu SP: **Cancer stem cell-like side population cells in clear cell renal cell carcinoma cell line 769P**. *PloS one* 2013, **8**(7):e68293.

7. Kozlowski JM, Fidler IJ, Campbell D, Xu ZL, Kaighn ME, Hart IR: **Metastatic behavior of human tumor cell lines grown in the nude mouse**. *Cancer research* 1984, **44**(8):3522-3529.

8. Veliceasa D, Ivanovic M, Hoepfner FT, Thumbikat P, Volpert OV, Smith ND: **Transient potential receptor channel 4 controls thrombospondin-1 secretion and angiogenesis in renal cell carcinoma**. *The FEBS journal* 2007, **274**(24):6365-6377.

9. **Gene Set - 769P ccle** [<http://amp.pharm.mssm.edu/Harmonizome/gene_set/769P/CCLE+Cell+Line+Gene+Mutation+Profiles>]

10. **Gene Set - 769-P cosmic** [<http://amp.pharm.mssm.edu/Harmonizome/gene_set/769-P/COSMIC+Cell+Line+Gene+Mutation+Profiles>]

11. Peña-Llopis S, Vega-Rubín-de-Celis S, Liao A, Leng N, Pavía-Jiménez A, Wang S, Yamasaki T, Zhrebker L, Sivanand S, Spence P *et al*: **BAP1 loss defines a new class of renal cell carcinoma**. *Nature Genetics* 2012, **44**:751-759.

12. Lee YH, Apolo AB, Agarwal PK, Bottaro DP: **Characterization of HGF/Met Signaling in Cell Lines Derived From Urothelial Carcinoma of the Bladder**. *Cancers (Basel)* 2014, **6**(4):2313-2329.

13. Balan M, Mier y Teran E, Waaga-Gasser AM, Gasser M, Choueiri TK, Freeman G, Pal S: **Novel roles of c-Met in the survival of renal cancer cells through the regulation of HO-1 and PD-L1 expression**. *The Journal of biological chemistry* 2015, **290**(13):8110-8120.

14. Kucejova B, Pena-Llopis S, Yamasaki T, Sivanand S, Tran TA, Alexander S, Wolff NC, Lotan Y, Xie XJ, Kabbani W *et al*: **Interplay between pVHL and mTORC1 pathways in clear-cell renal cell carcinoma**. *Molecular cancer research : MCR* 2011, **9**(9):1255-1265.

15. Shen C, Beroukhim R, Schumacher SE, Zhou J, Chang M, Signoretti S, Kaelin WG, Jr.: **Genetic and functional studies implicate HIF1alpha as a 14q kidney cancer suppressor gene**. *Cancer Discov* 2011, **1**(3):222-235.

16. Ding XF, Zhou J, Hu QY, Liu SC, Chen G: **The tumor suppressor pVHL down-regulates never-in-mitosis A-related kinase 8 via hypoxia-inducible factors to maintain cilia in human renal cancer cells**. *The Journal of biological chemistry* 2015, **290**(3):1389-1394.

17. Peruzzi B, Athauda G, Bottaro DP: **The von Hippel-Lindau tumor suppressor gene product represses oncogenic beta-catenin signaling in renal carcinoma cells**. *Proceedings of the National Academy of Sciences of the United States of America* 2006, **103**(39):14531-14536.

18. Ashida S, Nishimori I, Tanimura M, Onishi S, Shuin T: **Effects of von Hippel-Lindau gene mutation and methylation status on expression of transmembrane carbonic anhydrases in renal cell carcinoma**. *Journal of cancer research and clinical oncology* 2002, **128**(10):561-568.

19. Nakaigawa N, Yao M, Baba M, Kato S, Kishida T, Hattori K, Nagashima Y, Kubota Y: **Inactivation of von Hippel-Lindau gene induces constitutive phosphorylation of MET protein in clear cell renal carcinoma**. *Cancer research* 2006, **66**(7):3699-3705.

20. Boysen G, Bausch-Fluck D, Thoma CR, Nowicka AM, Stiehl DP, Cima I, Luu VD, von Teichman A, Hermanns T, Sulser T *et al*: **Identification and functional characterization of pVHL-dependent cell surface proteins in renal cell carcinoma**. *Neoplasia* 2012, **14**(6):535-546.

21. Lin Y, Yang Z, Xu A, Dong P, Huang Y, Liu H, Li F, Wang H, Xu Q, Wang Y *et al*: **PIK3R1 negatively regulates the epithelial-mesenchymal transition and stem-like phenotype of renal cancer cells through the AKT/GSK3β/CTNNB1 signaling pathway**. *Scientific reports* 2015, **5**:8997.

22. Zhang L, Jiao M, Wu K, Li L, Zhu G, Wang X, He D, Wu D: **TNF-alpha induced epithelial mesenchymal transition increases stemness properties in renal cell carcinoma cells**. *International journal of clinical and experimental medicine* 2014, **7**(12):4951-4958.

23. Liu YH, Lin CY, Lin WC, Tang SW, Lai MK, Lin JY: **Up-Regulation of Vascular Endothelial Growth Factor-D Expression in Clear Cell Renal Cell Carcinoma by CD74: A Critical Role in Cancer Cell Tumorigenesis**. *The Journal of Immunology* 2008, **181**(9):6584-6594.

24. Verhoest G, Dolley-Hitze T, Jouan F, Belaud-Rotureau MA, Oger E, Lavenu A, Bensalah K, Arlot-Bonnemains Y, Collet N, Rioux-Leclercq N *et al*: **Sunitinib combined with angiotensin-2 type-1 receptor antagonists induces more necrosis: a murine xenograft model of renal cell carcinoma**. *Biomed Res Int* 2014, **2014**:901371.

25. **Gene Set - 786-O** [<http://amp.pharm.mssm.edu/Harmonizome/gene_set/786-O/Klijn+et+al.%2C+Nat.+Biotechnol.%2C+2015+Cell+Line+Gene+Mutation+Profiles>]

26. Rouillard AD, Gundersen GW, Fernandez NF, Wang Z, Monteiro CD, McDermott MG, Ma’ayan A: **The harmonizome: a collection of processed datasets gathered to serve and mine knowledge about genes and proteins**. 2016.

27. **Gene Set - 786-0 COSMIC** [<http://amp.pharm.mssm.edu/Harmonizome/gene_set/786-0/COSMIC+Cell+Line+Gene+Mutation+Profiles>]

28. **Gene Set - 786O CCLE** [<http://amp.pharm.mssm.edu/Harmonizome/gene_set/786O/CCLE+Cell+Line+Gene+Mutation+Profiles>]

29. Giard DJ, Aaronson SA, Todaro GJ, Arnstein P, Kersey JH, Dosik H, Parks WP: **In vitro cultivation of human tumors: establishment of cell lines derived from a series of solid tumors**. *Journal of the National Cancer Institute* 1973, **51**(5):1417-1423.

30. Gemmill RM, Zhou M, Costa L, Korch C, Bukowski RM, Drabkin HA: **Synergistic growth inhibition by Iressa and Rapamycin is modulated by VHL mutations in renal cell carcinoma**. *Br J Cancer* 2005, **92**(12):2266-2277.

31. Gnarra JR, Tory K, Weng Y, Schmidt L, Wei MH, Li H, Latif F, Liu S, Chen F, Duh FM: **Mutations of the VHL tumour suppressor gene in renal carcinoma**. *Nature genetics* 1994, **7**(1):85-90.

32. Lovell M, Lott ST, Wong P, El-Naggar A, Tucker S, Killary AM: **The genetic locus NRC-1 within chromosome 3p12 mediates tumor suppression in renal cell carcinoma independently of histological type, tumor microenvironment, and VHL mutation**. *Cancer research* 1999, **59**(9):2182-2189.

33. Korhonen M, Sariola H, Gould VE, Kangas L, Virtanen I: **Integrins and laminins in human renal carcinoma cells and tumors grown in nude mice**. *Cancer research* 1994, **54**(16):4532-4538.

34. Huang D, Ding Y, Li Y, Luo W-M, Zhang Z-F, Snider J, Vandenbeldt K, Qian C-N, Teh BT: **Sunitinib acts primarily on tumor endothelium rather than tumor cells to inhibit the growth of renal cell carcinoma**. *Cancer research* 2010, **70**(3):1053-1062.

35. Huang D, Ding Y, Luo W-M, Bender S, Qian C-N, Kort E, Zhang Z-F, VandenBeldt K, Duesbery NS, Resau JH *et al*: **Inhibition of MAPK kinase signaling pathways suppressed renal cell carcinoma growth and angiogenesis in vivo**. *Cancer research* 2008, **68**(1):81-88.

36. Tomita Y, Bilim, V., Kawasaki, T., Takahashi, K., Okan, I., Magnusson, K. P., & Wiman, K. G.: **Frequent expression of Bcl‐2 in renal‐cell carcinomas carrying wild‐type p53.** . In: *International journal of cancer, 66(3), 322-325.* 1996.

37. Li W, Wang Q, Su Q, Ma D, An C, Ma L, Liang H: **Honokiol suppresses renal cancer cells' metastasis via dual-blocking epithelial-mesenchymal transition and cancer stem cell properties through modulating miR-141/ZEB2 signaling**. *Mol Cells* 2014, **37**(5):383-388.

38. **Gene Set - ACHN** [<http://amp.pharm.mssm.edu/Harmonizome/gene_set/ACHN/COSMIC+Cell+Line+Gene+Mutation+Profiles>]

39. **Gene Set - A498 ccle** [<http://amp.pharm.mssm.edu/Harmonizome/gene_set/A498/CCLE+Cell+Line+Gene+Mutation+Profiles>]

40. **Gene Set - A498 cosmic** [<http://amp.pharm.mssm.edu/Harmonizome/gene_set/A498/COSMIC+Cell+Line+Gene+Mutation+Profiles>]

41. Shen C, Beroukhim R, Schumacher SE, Zhou J, Chang M, Signoretti S, Kaelin WG: **Genetic and Functional Studies Implicate HIF1α as a 14q Kidney Cancer Suppressor Gene**. *Cancer Discov* 2011, **1**(3):222-235.

42. Ruf M, Mittmann C, Nowicka AM, Hartmann A, Hermanns T, Poyet C, Broek Mvd, Sulser T, Moch H, Schraml P: **pVHL/HIF-Regulated CD70 Expression Is Associated with Infiltration of CD27+ Lymphocytes and Increased Serum Levels of Soluble CD27 in Clear Cell Renal Cell Carcinoma**. 2015.

43. Gershwin ME, Lentz D, Owens RB: **Relationship between karyotype of tissue culture lines and tumorigenicity in nude mice**. *Exp Cell Biol* 1984, **52**(6):361-370.

44. Macher‐Goeppinger S, Germany UHHIoP, Germany GCRCDMTPH, Keith M, Germany UHHIoP, Germany GCRCDMTPH, Tagscherer KE, Germany UHHIoP, Germany GCRCDMTPH, Singer S *et al*: **PBRM1 (BAF180) protein is functionally regulated by p53‐induced protein degradation in renal cell carcinomas**. *The Journal of Pathology* 2016, **237**(4):460-471.

45. Borden EC, Hogan TF, Voelkel JG: **Comparative antiproliferative activity in vitro of natural interferons α and β for diploid and transformed human cells**, vol. 42; 1982.

46. Avnet S, Cenni E, Granchi D, Perut F, Amato I, Battistelli L, Brandi ML, Giunti A, Baldini N: **Isolation and characterization of a new cell line from a renal carcinoma bone metastasis**. *Anticancer research* 2004, **24**(3a):1705-1711.

47. Schmidt L, Junker K, Nakaigawa N, Kinjerski T, Weirich G, Miller M, Lubensky I, Neumann HP, Brauch H, Decker J *et al*: **Novel mutations of the MET proto-oncogene in papillary renal carcinomas**. *Oncogene* 1999, **18**(14):2343-2350.

48. Furge KA, Chen J, Koeman J, Swiatek P, Dykema K, Lucin K, Kahnoski R, Yang XJ, Teh BT: **Detection of DNA copy number changes and oncogenic signaling abnormalities from gene expression data reveals MYC activation in high-grade papillary renal cell carcinoma**. *Cancer research* 2007, **67**(7):3171-3176.

49. Kovacs G, Fuzesi L, Emanual A, Kung HF: **Cytogenetics of papillary renal cell tumors**. *Genes, chromosomes & cancer* 1991, **3**(4):249-255.

50. Hakimi AA, Chevinsky M, Hsieh JJ, Sander C, Sinha R: **Mp23-11 Genomic Comparison of Renal Cell Carcinoma Cell Lines to Human Tumors**. *The Journal of urology* 2014, **191**(4):e247.

51. Chang AY, Keng PC: **Inhibition of cell growth in synchronous human hypernephroma cells by recombinant interferon alpha-D and irradiation**. *Journal of interferon research* 1983, **3**(4):379-385.

52. Ueda K, Ogasawara S, Akiba J, Nakayama M, Todoroki K, Sanada S, Suekane S, Noguchi M, Matsuoka K, Yano H: **Aldehyde dehydrogenase 1 identifies cells with cancer stem cell-like properties in a human renal cell carcinoma cell line**. *PloS one* 2013, **8**(10):e75463.

53. Lichner Z, Saleh C, Subramaniam V, Seivwright A, Yousef GM: **Prud’homme, G. J., & miR-17 inhibition enhances the formation of kidney cancer spheres with stem cell/tumor initiating cell properties**. *Oncotarget 5* 2014.

54. Nishizawa S, Hirohashi Y, Torigoe T, Takahashi A, Tamura Y, Mori T, Kanaseki T, Kamiguchi K, Asanuma H, Morita R *et al*: **HSP DNAJB8 controls tumor-initiating ability in renal cancer stem-like cells**. *Cancer research* 2012, **72**(11):2844-2854.

55. Zhang L, Jiao M, Wu K, Li L, Zhu G, Wang X, He D, Wu D: **TNF-α induced epithelial mesenchymal transition increases stemness properties in renal cell carcinoma cells**. *International journal of clinical and experimental medicine* 2014, **7**(12):4951-4958.

56. Munnink THO, Tamas KR, de Hooge MN, Vedelaar SR, Timmer-Bosscha H, Walenkamp AM, de Vries EG: **Lub- ... & Placental growth factor (PlGF)–specific uptake in tumor microenvironment of 89Zr-labeled PlGF antibody RO5323441**. *Journal of Nuclear Medicine* 2013, **54**(6 SRC - GoogleScholar):929-935.

57. Wang L, Park P, La Marca F, Than KD, Lin CY: **BMP-2 inhibits tumor-initiating ability in human renal cancer stem cells and induces bone formation**. *Journal of cancer research and clinical oncology* 2015, **141**(6):1013-1024.

58. **Gene Set - ACHN CCLE** [<http://amp.pharm.mssm.edu/Harmonizome/gene_set/ACHN/CCLE+Cell+Line+Gene+Mutation+Profiles>]

59. **Gene Set - ACHN COSMIC** [<http://amp.pharm.mssm.edu/Harmonizome/gene_set/ACHN/COSMIC+Cell+Line+Gene+Mutation+Profiles>]

60. Varela I, Tarpey P, Raine K, Huang D, Ong CK, Stephens P, Davies H, Jones D, Lin M-L, Teague J *et al*: **Exome sequencing identifies frequent mutation of the SWI/SNF complex gene PBRM1 in renal carcinoma**. *Nature* 2011, **469**:539-542.

61. Chowdhury B, Porter EG, Stewart JC, Ferreira CR, Schipma MJ, Dykhuizen EC: **PBRM1 Regulates the Expression of Genes Involved in Metabolism and Cell Adhesion in Renal Clear Cell Carcinoma**. In: *PLoS One. Volume 11*, edn.; 2016.

62. Fogh J, Center MS-KC, Trempe G, Center MS-KC: **New Human Tumor Cell Lines**. 2016:115-159.

63. Fujisue Y, Nakagawa T, Takahara K, Inamoto T, Kiyama S, Azuma H, Asahi M: **Induction of erythropoietin increases the cell proliferation rate in a hypoxia-inducible factor-1-dependent and -independent manner in renal cell carcinoma cell lines**. *Oncol Lett* 2013, **5**(6):1765-1770.

64. Feng C, Sun Y, Ding G, Wu Z, Jiang H, Wang L, Ding Q, Wen H: **PI3Kbeta inhibitor TGX221 selectively inhibits renal cell carcinoma cells with both VHL and SETD2 mutations and links multiple pathways**. *Scientific reports* 2015, **5**:9465.

65. **Gene Set - CAKI1 ccle** [<http://amp.pharm.mssm.edu/Harmonizome/gene_set/CAKI1/CCLE+Cell+Line+Gene+Mutation+Profiles>]

66. Myers T, Chengedza S, Lightfoot S, Pan Y, Dedmond D, Cole L, Tang Y, Benbrook DM: **Flexible heteroarotinoid (Flex-Het) SHetA2 inhibits angiogenesis in vitro and in vivo**. *Invest New Drugs* 2009, **27**(4):304-318.

67. Lee HW, Joo KM, Lim JE, Cho HJ, Cho HJ, Park MC, Seol HJ, Seo SI, Lee J-I, Kim S *et al*: **Tpl2 kinase impacts tumor growth and metastasis of clear cell renal cell carcinoma**. *Molecular cancer research : MCR* 2013, **11**(11):1375-1386.

68. **Gene Set - CAKI-1 cosmic** [<http://amp.pharm.mssm.edu/Harmonizome/gene_set/CAKI-1/COSMIC+Cell+Line+Gene+Mutation+Profiles>]

69. Looyenga BD, Furge KA, Dykema KJ, Koeman J, Swiatek PJ, Giordano TJ, West AB, Resau JH, Teh BT, MacKeigan JP: **Chromosomal amplification of leucine-rich repeat kinase-2 (LRRK2) is required for oncogenic MET signaling in papillary renal and thyroid carcinomas**. *Proceedings of the National Academy of Sciences of the United States of America* 2011, **108**(4):1439-1444.

70. Pulkkanen KJ, Parkkinen JJ, Kettunen MI, Kauppinen RA, Lappalainen M, Ala-Opas MY, Yla-Herttuala S: **Characterization of a new animal model for human renal cell carcinoma**. *In vivo (Athens, Greece)* 2000, **14**(3):393-400.

71. **Gene Set - CAKI2 ccle** [<http://amp.pharm.mssm.edu/Harmonizome/gene_set/CAKI2/CCLE+Cell+Line+Gene+Mutation+Profiles>]

72. Graham FL, Smiley J, Russell WC, Nairn R: **Characteristics of a human cell line transformed by DNA from human adenovirus type 5**. *The Journal of general virology* 1977, **36**(1):59-74.

73. Hu CJ, Wang LY, Chodosh LA, Keith B, Simon MC: **Differential roles of hypoxia-inducible factor 1alpha (HIF-1alpha) and HIF-2alpha in hypoxic gene regulation**. *Mol Cell Biol* 2003, **23**(24):9361-9374.

74. Sakkab D, Lewitzky M, Posern G, Schaeper U, Sachs M, Birchmeier W, Feller SM: **Signaling of hepatocyte growth factor/scatter factor (HGF) to the small GTPase Rap1 via the large docking protein Gab1 and the adapter protein CRKL**. *The Journal of biological chemistry* 2000, **275**(15):10772-10778.

75. Stepanenko AA, Dmitrenko VV: **HEK293 in cell biology and cancer research: phenotype, karyotype, tumorigenicity, and stress-induced genome-phenotype evolution**. *Gene* 2015, **569**(2):182-190.

76. Shen C, Gu M, Song C, Miao L, Hu L, Liang D, Zheng C: **The tumorigenicity diversification in human embryonic kidney 293 cell line cultured in vitro**. *Biologicals : journal of the International Association of Biological Standardization* 2008, **36**(4):263-268.

77. Kwon HS, Shin HC, Kim JS: **Suppression of vascular endothelial growth factor expression at the transcriptional and post-transcriptional levels**. *Nucleic Acids Res* 2005, **33**(8):e74.

78. Lin Y-C, Boone M, Meuris L, Lemmens I, Roy NV, Soete A, Reumers J, Moisse M, Plaisance S, Drmanac R *et al*: **Genome dynamics of the human embryonic kidney 293 lineage in response to cell biology manipulations**. *Nature Communications, Published online: 3 September 2014; | doi:101038/ncomms5767* 2014.

79. Ryan MJ, Johnson G, Kirk J, Fuerstenberg SM, Zager RA, Torok-Storb B: **HK- an immortalized proximal tubule epithelial cell line from normal adult human kidney**. *Kidney Int 45* 1994, **2 SRC - GoogleScholar**:48-57.

80. Thapa RJ, Chen P, Cheung M, Nogusa S, Pei J, Peri S, Testa JR, Balachandran S: **NF-kappaB inhibition by bortezomib permits IFN-gamma-activated RIP1 kinase-dependent necrosis in renal cell carcinoma**. *Mol Cancer Ther* 2013, **12**(8):1568-1578.

81. Valera VA, Walter BA, Linehan WM, Merino MJ: **Regulatory Effects of microRNA-92 (miR-92) on VHL Gene Expression and the Hypoxic Activation of miR-210 in Clear Cell Renal Cell Carcinoma**. *J Cancer* 2011, **2**:515-526.

82. Naito K, Hisazumi H, Kanokogi M, Katoh M, Nakazima K, Tsukahara K, Kobayashi T, Kuroda K, Matsubara F: **[Establishment of cell lines (KH-39, KN-41, and KW-103) from human kidney and bladder cancer and their characteristics]**. *Nihon Hinyokika Gakkai zasshi The japanese journal of urology* 1982, **73**(8):1019-1031.

83. Yang Y, Kost-Alimova M, Ingvarsson S, Qianhui Q, Kiss H, Szeles A, Kholodnyuk I, Cuthbert A, Klein G, Imreh S: **Similar regions of human chromosome 3 are eliminated from or retained in human/human and human/mouse microcell hybrids during tumor growth in severe combined immunodeficient (SCID) mice**. *Proceedings of the National Academy of Sciences of the United States of America* 2001, **98**(3):1136-1141.

84. Alimov A, Kost-Alimova M, Liu J, Li C, Bergerheim U, Imreh S, Klein G, Zabarovsky ER: **Combined LOH/CGH analysis proves the existence of interstitial 3p deletions in renal cell carcinoma**. *Oncogene* 2000, **19**(11):1392-1399.

85. **Gene Set - KMRC2** [<http://amp.pharm.mssm.edu/Harmonizome/gene_set/KMRC2/CCLE+Cell+Line+Gene+Mutation+Profiles>]

86. Yano H, Maruiwa M, Sugihara S, Kojiro M, Noda S, Eto K: **Establishment and characterization of a new human renal cell carcinoma cell line (KRC/Y)**. *In vitro cellular & developmental biology : journal of the Tissue Culture Association* 1988, **24**(1):9-16.

87. Kinouchi T, Kotake T, Mori Y, Abe T: **Human renal cell carcinoma: establishment and characterization of a new cell line (OS-RC-2)**. *In Vitro Cell Dev Biol* 1985, **21**(4):195-199.

88. Xu K, Ding Q, Fang Z, Zheng J, Gao P, Lu Y, Zhang Y: **Silencing of HIF-1alpha suppresses tumorigenicity of renal cell carcinoma through induction of apoptosis**. *Cancer Gene Ther* 2010, **17**(3):212-222.

89. Xu Z, Shen G, Xia X, Zhao X, Zhang P, Wu H, Guo Q, Qian Z, Wei Y, Liang S: **Comparisons of three polyethyleneimine-derived nanoparticles as a gene therapy delivery system for renal cell carcinoma**. *J Transl Med* 2011, **9**:46.

90. Vanharanta S, Shu W, Brenet F, Hakimi AA, Heguy A, Viale A, Reuter VE, Hsieh JJ, Scandura JM, Massague J: **Epigenetic expansion of VHL-HIF signal output drives multiorgan metastasis in renal cancer**. *Nat Med* 2013, **19**(1):50-56.

91. Tsuchiya K, Hida K, Hida Y, Muraki C, Ohga N, Akino T, Kondo T, Miseki T, Nakagawa K, Shindoh M *et al*: **Adrenomedullin antagonist suppresses tumor formation in renal cell carcinoma through inhibitory effects on tumor endothelial cells and endothelial progenitor mobilization**. *Int J Oncol* 2010, **36**(6):1379-1386.

92. **Gene Set - OS-RC-2** [<http://amp.pharm.mssm.edu/Harmonizome/gene_set/OS-RC-2/COSMIC+Cell+Line+Gene+Mutation+Profiles>]

93. Razorenova OV, Finger EC, Colavitti R, Chernikova SB, Boiko AD, Chan CK, Krieg A, Bedogni B, LaGory E, Weissman IL *et al*: **VHL loss in renal cell carcinoma leads to up-regulation of CUB domain-containing protein 1 to stimulate PKC{delta}-driven migration**. *Proceedings of the National Academy of Sciences of the United States of America* 2011, **108**(5):1931-1936.

94. Harten SK, Esteban MA, Shukla D, Ashcroft M, Maxwell PH: **Inactivation of the von Hippel-Lindau tumour suppressor gene induces Neuromedin U expression in renal cancer cells**. *Mol Cancer* 2011, **10**:89.

95. Doonachar A, Gallo MD, Doukas D, Pasricha R, Lantsberg I, Schoenfeld AR: **Differential effects of HIF-alpha isoforms on apoptosis in renal carcinoma cell lines**. *Cancer Cell Int* 2015, **15**:23.

96. Esteban MA, Tran MG, Harten SK, Hill P, Castellanos MC, Chandra A, Raval R, O'Brien T S, Maxwell PH: **Regulation of E-cadherin expression by VHL and hypoxia-inducible factor**. *Cancer research* 2006, **66**(7):3567-3575.

97. Krieg M, Haas R, Brauch H, Acker T, Flamme I, Plate KH: **Up-regulation of hypoxia-inducible factors HIF-1alpha and HIF-2alpha under normoxic conditions in renal carcinoma cells by von Hippel-Lindau tumor suppressor gene loss of function**. *Oncogene* 2000, **19**(48):5435-5443.

98. Viey E, Fromont G, Escudier B, Morel Y, Da Rocha S, Chouaib S, Caignard A: **Phosphostim-Activated    T Cells Kill Autologous Metastatic Renal Cell Carcinoma**. *The Journal of Immunology* 2005, **174**(3):1338-1347.

99. Gati A, Da Rocha S, Guerra N, Escudier B, Moretta A, Chouaib S, Angevin E, Caignard A: **Analysis of the natural killer mediated immune response in metastatic renal cell carcinoma patients**. *International journal of cancer* 2004, **109**(3):393-401.

100. Perier A, Fregni G, Wittnebel S, Gad S, Allard M, Gervois N, Escudier B, Azzarone B, Caignard A: **Mutations of the von Hippel-Lindau gene confer increased susceptibility to natural killer cells of clear-cell renal cell carcinoma**. *Oncogene* 2011, **30**(23):2622-2632.

101. Guerra N, Michel F, Gati A, Gaudin C, Mishal Z, Escudier B, Acuto O, Chouaib S, Caignard A: **Engagement of the inhibitory receptor CD158a interrupts TCR signaling, preventing dynamic membrane reorganization in CTL/tumor cell interaction**. *Blood* 2002, **100**(8):2874-2881.

102. Thrash-Bingham CA, Salazar H, Freed JJ, Greenberg RE, Tartof KD: **Genomic alterations and instabilities in renal cell carcinomas and their relationship to tumor pathology**. *Cancer research* 1995, **55**(24):6189-6195.

103. Li L, Zhang L, Zhang X, Yan Q, Minamishima YA, Olumi AF, Mao M, Bartz S, Kaelin WG, Jr.: **Hypoxia-inducible factor linked to differential kidney cancer risk seen with type 2A and type 2B VHL mutations**. *Mol Cell Biol* 2007, **27**(15):5381-5392.

104. Maxwell PH, Wiesener MS, Chang GW, Clifford SC, Vaux EC, Cockman ME, Wykoff CC, Pugh CW, Maher ER, Ratcliffe PJ: **The tumour suppressor protein VHL targets hypoxia-inducible factors for oxygen-dependent proteolysis**. *Nature* 1999, **399**(6733):271-275.

105. Ranieri E, Battaglia M, Wolfgang H, Gesualdo L, Bari UDSD, Foggia UDSD: **Renal carcinoma cell line and uses thereof**. 2012.

106. **Gene Set - RCC-ER** [<http://amp.pharm.mssm.edu/Harmonizome/gene_set/RCC-ER/COSMIC+Cell+Line+Gene+Mutation+Profiles>]

107. Duns G, Berg Evd, Duivenbode Iv, Osinga J, Hollema H, Hofstra RMW, Kok K: **Histone Methyltransferase Gene SETD2 Is a Novel Tumor Suppressor Gene in Clear Cell Renal Cell Carcinoma**. 2010.

108. Hogemann I, Bock S, Heppner P, Petrides PE: **Cytogenetic and growth factor gene analysis of a renal carcinoma cell line**. *Cancer genetics and cytogenetics* 1994, **78**(2):175-180.

109. Morris MR, Hughes DJ, Tian YM, Ricketts CJ, Lau KW, Gentle D, Shuib S, Serrano-Fernandez P, Lubinski J, Wiesener MS *et al*: **Mutation analysis of hypoxia-inducible factors HIF1A and HIF2A in renal cell carcinoma**. *Anticancer research* 2009, **29**(11):4337-4343.

110. Gross AJ, Wolff M, Fandrey J, Miersch WD, Dieckmann KP, Jelkmann W: **Prevalence of paraneoplastic erythropoietin production by renal cell carcinomas**. *The Clinical investigator* 1994, **72**(5):337-340.

111. Baba M, Hirai S, Kawakami S, Kishida T, Sakai N, Kaneko S, Yao M, Shuin T, Kubota Y, Hosaka M *et al*: **Tumor suppressor protein VHL is induced at high cell density and mediates contact inhibition of cell growth**. *Oncogene* 2001, **20**(22):2727-2736.

112. Simon BR, Wilson MJ, Wickliffe JK: **The RPTEC/TERT1 cell line models key renal cell responses to the environmental toxicants, benzo[a]pyrene and cadmium**. *Toxicol Rep* 2014, **1**:231-242.

113. Wieser M, Stadler G, Jennings P, Streubel B, Pfaller W, Ambros P, Riedl C, Katinger H, Grillari J, Grillari-Voglauer R: **hTERT alone immortalizes epithelial cells of renal proximal tubules without changing their functional characteristics**. *American journal of physiology Renal physiology* 2008, **295**(5):F1365-1375.

114. Ebert T, Bander NH, Finstad CL, Ramsawak RD, Old LJ: **Establishment and characterization of human renal cancer and normal kidney cell lines**. *Cancer research* 1990, **50**(17):5531-5536.

115. da Silva NF, Gentle D, Hesson LB, Morton DG, Latif F, Maher ER: **Analysis of the Birt-Hogg-Dube (BHD) tumour suppressor gene in sporadic renal cell carcinoma and colorectal cancer**. *Journal of medical genetics* 2003, **40**(11):820-824.

116. Grabmaier K, MC AdW, Verhaegh GW, Schalken JA, Oosterwijk E: **Strict regulation of CAIX(G250/MN) by HIF-1alpha in clear cell renal cell carcinoma**. *Oncogene* 2004, **23**(33):5624-5631.

117. Sjolund J, Johansson M, Manna S, Norin C, Pietras A, Beckman S, Nilsson E, Ljungberg B, Axelson H: **Suppression of renal cell carcinoma growth by inhibition of Notch signaling in vitro and in vivo**. *J Clin Invest* 2008, **118**(1):217-228.

118. Miyao N, Tsukamoto T, Kumamoto Y: **Establishment of three human renal cell carcinoma cell lines (SMKT-R- SMKT-R-2, and SMKT-R-3) and their characters**. *Urol Res 17* 1989, **1 SRC - GoogleScholar**:317-324.

119. Tochizawa S, Masumori N, Yanai Y, Ohmoto Y, Yabuuchi Y, Tsukamoto T: **Antitumor effects of a combination of interferon-alpha and sorafenib on human renal carcinoma cell lines**. *Biomedical research (Tokyo, Japan)* 2008, **29**(6):271-278.

120. Tanaka T, Torigoe T, Hirohashi Y, Sato E, Honma I, Kitamura H, Masumori N, Tsukamoto T, Sato N: **Hypoxia-inducible factor (HIF)-independent expression mechanism and novel function of HIF prolyl hydroxylase-3 in renal cell carcinoma**. *Journal of cancer research and clinical oncology* 2014, **140**(3):503-513.

121. Kobayashi T, Honke K, Miyazaki T, Matsumoto K, Nakamura T, Ishizuka I, Makita A: **Hepatocyte growth factor specifically binds to sulfoglycolipids**. *The Journal of biological chemistry* 1994, **269**(13):9817-9821.

122. Thomas GV, Tran C, Mellinghoff IK, Welsbie DS, Chan E, Fueger B, Czernin J, Sawyers CL: **Hypoxia-inducible factor determines sensitivity to inhibitors of mTOR in kidney cancer**. *Nat Med* 2006, **12**(1):122-127.

123. Sanchez Y, el-Naggar A, Pathak S, Killary AM: **A tumor suppressor locus within 3p14-p12 mediates rapid cell death of renal cell carcinoma in vivo**. *Proceedings of the National Academy of Sciences of the United States of America* 1994, **91**(8):3383-3387.

124. Turcotte S, Chan DA, Sutphin PD, Hay MP, Denny WA, Giaccia AJ: **A molecule targeting VHL-deficient renal cell carcinoma that induces autophagy**. *Cancer Cell* 2008, **14**(1):90-102.

125. **Gene Set - SN12C** [<http://amp.pharm.mssm.edu/Harmonizome/gene_set/SN12C/COSMIC+Cell+Line+Gene+Mutation+Profiles>]

126. Shin KH, Ku JL, Kim WH, Lee SE, Lee C, Kim SW, Park JG: **Establishment and characterization of seven human renal cell carcinoma cell lines**. *BJU international* 2000, **85**(1):130-138.

127. **Gene Set - SNU1272** [<http://amp.pharm.mssm.edu/Harmonizome/gene_set/SNU1272/CCLE+Cell+Line+Gene+Mutation+Profiles>]

128. **Gene Set - SNU349** [<http://amp.pharm.mssm.edu/Harmonizome/gene_set/SNU349/CCLE+Cell+Line+Gene+Mutation+Profiles>]

129. Plonowski A, Schally AV, Nagy A, Kiaris H, Hebert F, Halmos G: **Inhibition of metastatic renal cell carcinomas expressing somatostatin receptors by a targeted cytotoxic analogue of somatostatin AN-238**. *Cancer Res* 2000, **60**(11):2996-3001.

130. Bear A, Clayman RV, Elbers J, Limas C, Wang N, Stone K, Gebhard R, Prigge W, Palmer J: **Characterization of two human cell lines (TK-10, TK-164) of renal cell cancer**. *Cancer research* 1987, **47**(14):3856-3862.

131. Lou F, Chen X, Jalink M, Zhu Q, Ge N, Zhao S, Fang X, Fan Y, Björkholm M, Liu Z *et al*: **The opposing effect of hypoxia-inducible factor-2alpha on expression of telomerase reverse transcriptase**. *Molecular cancer research : MCR* 2007, **5**(8):793-800.

132. LaGory EL, Wu C, Taniguchi CM, Ding CK, Chi JT, von Eyben R, Scott DA, Richardson AD, Giaccia AJ: **Suppression of PGC-1alpha Is Critical for Reprogramming Oxidative Metabolism in Renal Cell Carcinoma**. *Cell Rep* 2015, **12**(1):116-127.

133. **Gene Set - TK10** [<http://amp.pharm.mssm.edu/Harmonizome/gene_set/TK10/COSMIC+Cell+Line+Gene+Mutation+Profiles>]

134. Grossman HB, Wedemeyer G, Ren LQ: **Human renal carcinoma: characterization of five new cell lines**. *Journal of surgical oncology* 1985, **28**(3):237-244.

135. Kuzmin I, Geil L, Ge H, Bengtsson U, Duh FM, Stanbridge EJ, Lerman MI: **Analysis of aberrant methylation of the VHL gene by transgenes, monochromosome transfer, and cell fusion**. *Oncogene* 1999, **18**(41):5672-5679.

136. Martin-Puig S, Temes E, Olmos G, Jones DR, Aragones J, Landazuri MO: **Role of iron (II)-2-oxoglutarate-dependent dioxygenases in the generation of hypoxia-induced phosphatidic acid through HIF-1/2 and von Hippel-Lindau-independent mechanisms**. *The Journal of biological chemistry* 2004, **279**(10):9504-9511.

137. Anglard P, Trahan E, Liu S, Latif F, Merino MJ, Lerman MI, Zbar B, Linehan WM: **Molecular and cellular characterization of human renal cell carcinoma cell lines**. *Cancer research* 1992, **52**(2):348-356.

138. Herman JG, Latif F, Weng Y, Lerman MI, Zbar B, Liu S, Samid D, Duan DS, Gnarra JR, Linehan WM: **Silencing of the VHL tumor-suppressor gene by DNA methylation in renal carcinoma**. *Proceedings of the National Academy of Sciences of the United States of America* 1994, **91**(21):9700-9704.

139. Reiter RE, Anglard P, Liu S, Gnarra JR, Linehan WM: **Chromosome 17p deletions and p53 mutations in renal cell carcinoma**. *Cancer research* 1993, **53**(13):3092-3097.

140. Pavlovich CP, Padilla-Nash H, Wangsa D, Nickerson ML, Matrosova V, Linehan WM, Ried T, Phillips JL: **Patterns of aneuploidy in stage IV clear cell renal cell carcinoma revealed by comparative genomic hybridization and spectral karyotyping**. *Genes, chromosomes & cancer* 2003, **37**(3):252-260.

141. Alleman WG, Tabios RL, Chandramouli GV, Aprelikova ON, Torres-Cabala C, Mendoza A, Rogers C, Sopko NA, Linehan WM, Vasselli JR: **The in vitro and in vivo effects of re-expressing methylated von Hippel-Lindau tumor suppressor gene in clear cell renal carcinoma with 5-aza-2'-deoxycytidine**. *Clin Cancer Res* 2004, **10**(20):7011-7021.

142. Sourbier C, Srivastava G, Ghosh MC, Ghosh S, Yang Y, Gupta G, Degraff W, Krishna MC, Mitchell JB, Rouault TA *et al*: **Targeting HIF2alpha translation with Tempol in VHL-deficient clear cell renal cell carcinoma**. *Oncotarget* 2012, **3**(11):1472-1482.

143. An J, Liu H, Magyar CE, Guo Y, Veena MS, Srivatsan ES, Huang J, Rettig MB: **Hyperactivated JNK is a therapeutic target in pVHL-deficient renal cell carcinoma**. *Cancer research* 2013, **73**(4):1374-1385.

144. Clark J, Lu YJ, Sidhar SK, Parker C, Gill S, Smedley D, Hamoudi R, Linehan WM, Shipley J, Cooper CS: **Fusion of splicing factor genes PSF and NonO (p54nrb) to the TFE3 gene in papillary renal cell carcinoma**. *Oncogene* 1997, **15**(18):2233-2239.

145. Mathur M, Das S, Samuels HH: **PSF-TFE3 oncoprotein in papillary renal cell carcinoma inactivates TFE3 and p53 through cytoplasmic sequestration**. *Oncogene* 2003, **22**(32):5031-5044.

146. Zhong M, Weisman P, Zhu B, Brassesco M, Yang Y, Linehan WM, Merino MJ, Zhang D, Rohan S, Cai D *et al*: **Xp11.2 translocation renal cell carcinoma with PSF-TFE3 rearrangement**. *Diagnostic molecular pathology : the American journal of surgical pathology, part B* 2013, **22**(2):107-111.

147. Jia LQ, Institute of Development AaC, Tohoku University, Sendai, Japan, China–Japan Friendship Hospital B, China, Osada M, Institute of Development AaC, Tohoku University, Sendai, Japan, Ishioka C, Institute of Development AaC, Tohoku University, Sendai, Japan, Institute of Development AaC, Tohoku University, 4‐1 Seiryo‐machi, Aoba‐ku, Sendai 980, Japan, Gamo M, Institute of Development AaC, Tohoku University, Sendai, Japan *et al*: **Screening the p53 status of human cell lines using a yeast functional assay**. *Molecular Carcinogenesis* 2016, **19**(4):243-253.

148. Mengelbier LH, Bexell D, Sehic D, Ciornei CD, Gisselsson D: **Orthotopic Wilms tumor xenografts derived from cell lines reflect limited aspects of tumor morphology and clinical characteristics**. *Pediatric blood & cancer* 2014, **61**(11):1949-1954.
